# Isolated Rhodium Atoms Activate Porous TiO_2_ for Enhanced Electrocatalytic Conversion of Nitrate to Ammonia

**DOI:** 10.1002/advs.202411705

**Published:** 2024-11-18

**Authors:** Zhi Liang Zhao, Shaoxuan Yang, Shensong Wang, Zhe Zhang, Liang Zhao, Qi Wang, Xinyi Zhang

**Affiliations:** ^1^ National energy key laboratory for new hydrogen‐ammonia energy technologies Foshan Xianhu Laboratory Foshan 528200 P. R. China; ^2^ Hubei Key Laboratory of Micro‐Nanoelectronic Materials and Devices School of Microelectronics Hubei University Wuhan 430062 P. R. China; ^3^ College of Physics Science and Technology & Microelectronics Industry Research Institute Yangzhou University Jiangsu 225009 P. R. China; ^4^ Department of Materials Science and Engineering City University of Hong Kong Kowloon Hong Kong 999077 P. R. China

**Keywords:** ammonia synthesis, electrocatalysis, nitrate reduction reaction, single atom catalysts

## Abstract

The direct electrochemical reduction of nitrate to ammonia is an efficient and environmentally friendly technology, however, developing electrocatalysts with high activity and selectivity remains a great challenge. Single‐atom catalysts demonstrate unique properties and exceptional performance across a range of catalytic reactions, especially those that encompass multi‐step processes. Herein, a straightforward and cost‐effective approach is introduced for synthesizing single‐atom dispersed Rh on porous TiO_2_ spheres (Rh_1_‐TiO_2_), which functions as an efficient electrocatalyst for the electroreduction of NO_3_^−^ to NH_3_. The synthesized Rh_1_‐TiO_2_ catalyst achieve a maximum NH_3_ Faradaic efficiency (FE) of 94.7% and an NH_3_ yield rate of 29.98 mg h^−1^ mg_cat_^−1^ at −0.5 V versus RHE in a 0.1 m KOH+0.1 m KNO_3_ electrolyte, significantly outperforming not only undoped TiO_2_ but also Ru, Pd, and Ir single‐atom doped titania catalysts. Density functional theory calculations reveal that the incorporation of Rh single atom significantly enhances charge transfer between adsorbed NO_3_^−^ and the active site. The Rh atoms not only serve as the highly active site for electrochemical nitrate reduction reaction (NO_3_RR), but also activates the adjacent Ti sites through optimizating the electronic structure, thereby reducing the energy barrier of the rate‐limiting step. Consequently, this results in a substantial enhancement in electrochemical NO_3_RR performance. Furthermore, this synthetic method has the potential to be extended to other single‐atom catalysts and scaled up for commercial applications.

## Graphical abstract

A simplified spray‐drying technique is developed for large‐scale preparation of Rh single‐atoms anchored on nanoporous TiO_2_ spheres. The atomically dispersed Rh atoms serve as highly active catalytic sites for electrochemical nitrate reduction, while also activating adjacent Ti sites via optimization of their electronic structure. This subsequently reduces the energy barrier for the rate‐limiting step.

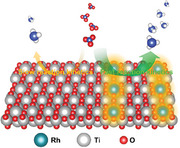

## 1 Introduction

Ammonia is a potential carbon‐free energy carrier, and boasts a number of advantages including high energy density, ease of liquefaction and storage, high safety, and a comprehensive transportation system, thus being regarded as one of the most promising clean fuels for the future.^[^ ^1^ ^]^ Traditional ammonia synthesis via the Haber–Bosch process involves reacting nitrogen separated from air with hydrogen obtained from fossil fuel reforming under high‐temperature and high‐pressure conditions, resulting in significant carbon emissions and high energy consumption.^[^ ^2^ ^]^ Consequently, green ammonia synthesis has become a coveted objective. The electrochemical method possesses the advantages of mild reaction conditions, selective conversion, and renewable energy utilization, rendering it a potentially promising technology for future applications. Recently, inspired by natural microbial nitrogen fixation, the electrochemical reduction of nitrogen to ammonia (NRR) has garnered significant attention.^[^ ^3^ ^]^ Nevertheless, the robust N≡N bond (941 kJ moL^−1^) and the poor solubility of nitrogen in aqueous media result in a poor ammonia yield rates and a low FE, impeding its widespread commercialization.^[^ ^4^ ^]^ Alternatively, nitrate (NO_3_^−^) is a more suitable nitrogen source for electrosynthesis of NH_3_ due to NO_3_^−^ dissociated into deoxygenated species requires a significantly lower energy (204 kJ moL^−1^) in comparison of NRR.^[^ ^5^ ^]^ On the other hand, excessive human interference has led to a yearly increase in nitrate levels in surface and groundwater, making the treatment of excessive nitrates in water bodies a crucial task for ecological environments and human health.^[^ ^6^ ^]^ Consequently, utilizing nitrate pollutants as a nitrogen source, electrochemical reduction of nitrate to ammonia selectively under mild conditions presents a promising avenue for simultaneously addressing energy and environmental challenges. However, the electrocatalytic reduction of NO_3_^−^ to NH_3_ is a highly complex chemical reaction that involves multiple steps, this reaction entails an intricate nine‐proton and eight‐electron transfer process, encompassing diverse reaction pathways and intermediate species, and competition with the hydrogen evolution reaction (HER).^[^ ^5^, ^7^ ^]^ Therefore, the development and scale‐up of high‐performance catalysts possessing superior activity and selectivity are in high demand.

Earlier studies on electrocatalytic nitrate reduction primarily centered on polarographic study, which highlights the significance of d‐orbital electrons in facilitating charge injection into the lowest unoccupied molecular orbital of nitrate.^[^ ^8^ ^]^ Despite the reported use of various noble metal‐based electrocatalysts, including Ir, Pd, Ru, Ag, and Au for NO_3_^−^ electroreduction,^[^ ^9^ ^]^ however, the application of pure metal electrocatalysts remains limited due to their underwhelming performance, attributed to the weak adsorption of nitrogen‐containing intermediates on their surfaces.^[^ ^7^, ^10^ ^]^ Additionally, the prohibitively high costs and limited reserves of noble metals hinder their widespread use as catalysts, there is a pressing need to minimize the noble metal content in electrodes while maintaining their catalytic activity. Consequently, developing an effective strategy to modulate the electronic structure is paramount in enhancing the inherent activity of pristine catalysts.^[^ ^11^ ^]^ Owing to its maximized atomic utilization, adjustable metal centers, diverse coordination environments, and reduced the usage of precious metals, single‐atom catalysts have garnered significant attention in the realm of electrocatalysis, including CO_2_ reduction,^[^ ^12^ ^]^ hydrogen evolution,^[^ ^13^ ^]^ and oxygen reduction reactions.^[^ ^14^ ^]^ Their key advantage stems from the fully exposed active sites, facilitating strong interactions with neighboring coordination atoms, thereby affording exceptional activity and stability to the single‐atom catalysts.^[^ ^15^ ^]^ Metal oxides are commonly utilized as supports for stabilizing single atoms and increasing surface‐exposed sites, owing to their high stability, non‐toxicity, and ubiquitous availability.^[^ ^16^ ^]^ Due to its high stability, eco‐friendly, and abundant characteristics, TiO_2_ has been considered as a promising material for a wide range of applications including NO_3_RR, and can serve as a support for dispersing high‐activity transition metal‐based materials.^[^ ^17^ ^]^ Especially, TiO_2_ can serve as an excellent substrate for noble metal single‐atom catalysts.

Spray drying is a well‐known method for preparing granular materials, which can be easily scale‐up and offers a more efficient means to obtain practical yields. In this work, we report a facile spray‐drying method to synthesize highly dispersed single‐atom precious metalson nanoporous TiO_2_ spheres as catalysts toward electroreduction of NO_3_^−^ to NH_3_. Leveraging the benefits of the single‐atom catalyst structure, we significantly minimize the precious metal usage in the catalyst while enhancing its electrochemical activity for nitrate reduction. Notably, the single‐atom dispersed Rh on porous TiO_2_ spheres (Rh_1_‐TiO_2_) demonstrated superior electrochemical activity and selectivity, achieving a FE of 94.7% at −0.5 V versus RHE, which surpasses that of pristine titania and Ru, Pd, and Ir single‐atom doped titania catalysts. Density functional theory (DFT) calculations revealed that the single‐atom Rh sites not only functioned as high‐performance catalytic active sites, but also activating the adjacent Ti sites, ultimately resulting in high catalytic activity and selectivity for NO_3_RR.

## 2 Results and Discussion

**Figure 1:**
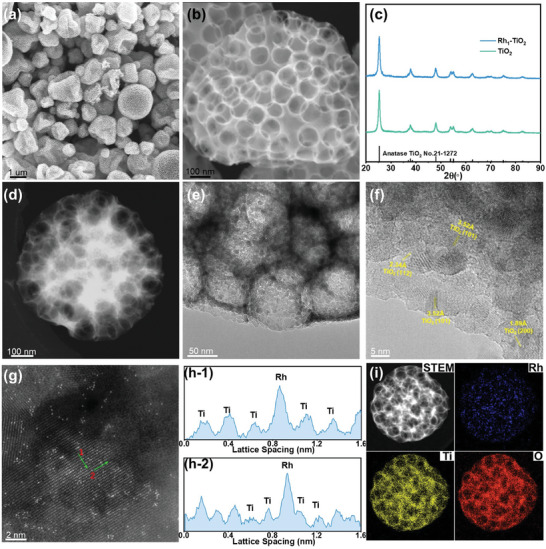
a) Scanning electron microscopy (SEM) image of the as‐synthesized Rh_1_‐TiO_2_ nanoparticles. b) Representative SEM image of a Rh_1_‐TiO_2_ nanoparticle. c) X‐ray diffraction (XRD) patterns of Rh_1_‐TiO_2_ and TiO_2_, including reference peak positions of anatase TiO_2_ (JCPDS file No.21‐1272). d) Scanning transmission Electron Microscopy (STEM) images of a representative Rh_1_‐TiO_2_ nanoparticle. e) Enlarged TEM image revealing a distinct foam‐like structure of the Rh_1_‐TiO_2_ particle. f) HRTEM image of the Rh_1_‐TiO_2_. g) Representative High‐Angle Annular Dark‐Field Scanning Transmission Electron Microscopy (HAADF‐STEM) image of the Rh_1_‐TiO_2_, in which the bright spots are attributed to Rh single atoms. h) The line profiles for HAADF intensity analysis, as labeled in Figure (g). i) HAADF‐STEM image of a representative Rh_1_‐TiO_2_ nanoparticle and the corresponding EDX elemental distribution mapping of Rh(i‐1), Ti(i‐2), and O(i‐3).

The synthesis procedure of Rh_1_‐TiO_2_ catalysts are as depicted in Figure S1 (Supporting Information). Initially, the monodispersed PMMA microspheres are used as hard template dispersed solution together with noble metal salt and titanium oxide precursor. Subsequently, the spray‐drying process ensures this solution forms droplets, in which the latex beads self‐assemble through an evaporation‐assisted process, the noble metal uniform dispersed with titanium oxide species uniform and precipitate around the PMMA spheres forming organic–inorganic hybrid spheroidal structures. Following pyrolysis in air, the PMMA polymeric template is removed and formed a nanoporous TiO_2_ network supported noble metal single atoms. During this process, PMMA microspheres serve as the microparticle template, while noble metal salts and TiCl_4_ serve as the precursors for single‐atom noble metals and TiO_2_, respectively. Spray drying is an efficient and versatile method for material preparation that can be easily scaled up to meet to industrial production requirements. Figure S2 (Supporting Information) shows the SEM images of the as‐prepared PMMA microspheres, the microspheres exhibit excellent size uniformity, having a diameter consistently approximating 110 nanometers. **Figure** **1**a,b shows the SEM images of the Rh_1_‐TiO_2_ spheres prepared by spray‐drying followed with a pyrolysis process in air, its sizes are ranged from 400 nm to 3 um. The spheres are hollow (Figure S3a,b, Supporting Information) with a thin shell and uniformly distributed with pores throughout the shell, stemming from the utilization of highly monodisperse PMMA microspheres as template.

The X‐ray diffraction (XRD) patterns of the as‐prepared porous TiO_2_ and Rh_1_‐TiO_2_ are shown in Figure 1c, the diffraction peaks of the two catalysts are well matched with the anatase TiO_2_ structure (JCPDS No.21‐1272), there is no any impurity peak of metallic rhodium or rhodium oxides observed. The calculated grains size is ≈7.8 nm according to the Debye‐Scherrer equation. It is noteworthy that the diffraction peak position of Rh_1_‐TiO_2_ shows almost no shift compared to that of TiO_2_, this suggests that Rh atoms are primarily located on the surface rather than incorporated into the TiO_2_ lattice, resulting in no significant changes to the lattice constants.^[^ ^14a^ ^]^ Figure 1d shows the STEM image of a representative Rh_1_‐TiO_2_ nanoparticle with a diameter of 650 nm. The particle holds a distinct foam‐like structure and inverse opal morphology with a pore opening diameter of 85 nm (Figure S3c, Supporting Information), indicating a shrinkage of the initial pores obtained by the 110 nm PMMA beads by ≈23% during the pyrolysis process. Figure 1e displays an enlarged TEM image of the Rh_1_‐TiO_2_ nanoparticle, the nanoparticle exhibits a nano‐polycrystalline structure composed of small nanograins. Figure 1f presents the high‐resolution TEM (HR‐STEM) image of the Rh_1_‐TiO_2_ nanoparticle, which further confirms the polycrystalline structure with an average grains size of ≈7.3 nm, the size in agreement with the XRD results calculated by using the Debye‐Scherrer equation. The lattice spacings of 3.52, 2.34, and 1.89 Å can be attributed to the (101), (112), and (200) planes of anatase TiO_2_, respectively, among these planes, the (101) plane is the most dominant.

The aberration−corrected high−angle annular darkfield scanning transmission electron microscopy (HAADF−STEM) image in Figure 1g shows that the bright Rh single atoms (the HAADF−STEM images reflect the Z‐contrast of materials, the Rh atoms have an intensity higher than that of the Ti atoms) are atomically dispersed on the crystal lattice of TiO_2_. Figure 1h shows the atomic line profiles in Figure 1g, in which bright contrast spots are identified as Rh atoms. The Rh atoms are located at exactly the same columns of Ti atoms, suggesting the Rh atoms are exclusively located at the original Ti position.^[^ ^14a^ ^]^ No metallic Rh or rhodium oxide particles were observed through HRTEM, consistent with the absence of metallic Rh or rhodium oxide diffraction peaks in the XRD pattern, further confirming the Rh are atomically dispersed on TiO_2_. The elemental mappings of Rh, Ti, and O in a representative Rh_1_‐TiO_2_ nanoparticle are depicted in Figure 1i, the Rh is uniform distribution on the whole nanoparticle. The Rh content in the product was calculated to be 1.08 at.% from the EDS mapping, which is in good agreement with the ICP‐AES results (Table S1, Supporting Information). The loading of rhodium within the materials can be adjusted by varying the number of Rh precursors. At a rhodium loading of ≈2 at.% (Rh_2_‐TiO_2_), both Rh single atoms and a substantial number of clusters were observed (Figure S4a, Supporting Information). In contrast, at a Rh loading of ≈0.5 at.% (Rh_0.5_‐TiO_2_), while all rhodium was present as single atoms, but their density was markedly lower compared to that at ≈1 at.% one. (Figure S4b, Supporting Information). Furthermore, the versatility of our novel preparation method extends beyond the initial synthesis, demonstrating broad applicability in the field of other single‐atom catalysis. We have successfully applied this technique to synthesize a range of noble metal single‐atom catalysts, including Ru, Pd, and Ir all dispersed on porous support materials (Figures S5 and S6, Supporting Information). The successful adaptability of the method to various noble metals underscores its flexibility and promising potential for broad implementation in catalyst synthesis.

**Figure 2:**
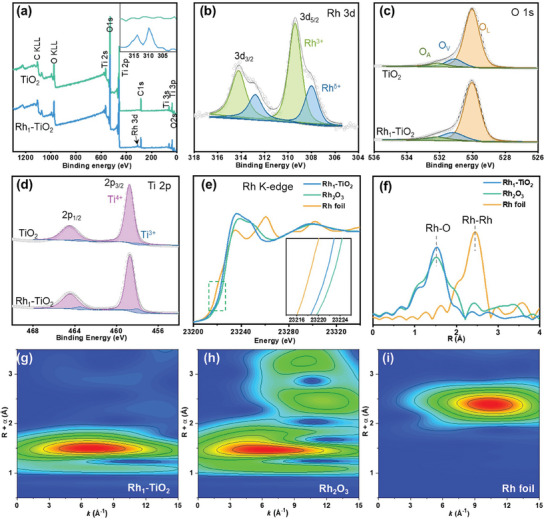
a) Full‐scan XPS spectrum of Rh_1_‐TiO_2_ and TiO_2_. High‐resolution XPS spectra of b) Rh 3d, c) O 1s, d) Ti 2p. e)XANES spectra and f) FT‐EXAFS spectra of Rh_1_‐TiO_2_, Rh foil, and Rh_2_O_3_ at the Rh K‐edge. k^3^‐weighted WT‐EXAFS spectra of g) Rh_1_‐TiO_2_, h) Rh_2_O_3_ and i) Rh foil.

The valences and chemical states of the as‐prepared Rh_1_‐TiO_2_ catalyst were evaluated by using X‐ray photoelectron spectroscopy (XPS) and synchrotron X‐ray absorption spectroscopy (XAS). The full‐scan spectra of Rh_1_‐TiO_2_ and TiO_2_ shown in **Figure** **2**a indicate that C, O, and Ti can be identified. The carbon species may originate from the impurities during the material synthesis or atmospheric adsorption on the sample surface. These can serve as an internal reference, with a binding energy value of 284.6 eV assigned to compensate for charging effects.^[^ ^18^ ^]^ In comparison to TiO_2_, two additional weak peaks located at binding energies of 310 and 315 eV (inset of Figure 2a) can be assigned to Rh 3d_5/2_ and 3d_3/2_ core levels, respectively. The XPS analysis indicates a Rh content of 2.25 at.%, which significantly exceeding the EDS and ICP‐AES results. As XPS is a surface‐sensitive detection technology, the higher Rh content observed suggests enrichment of Rh on the surface of the material,^[^ ^19^ ^]^ which aligns with the XRD findings. To further investigate the chemical states of Rh in Rh_1_‐TiO_2_ catalyst, the high‐resolution XPS spectroscopy of Rh 3d core‐levels is presented in Figure 2b. The Rh 3d core‐levels split into 3d_5/2_ and 3d_3/2_ states due to the spin‐orbital coupling. The binding energies of the peak located at 309.3 and 314.0 eV correspond to Rh 3d_5/2_ and Rh 3d_3/2_, respectively, which can be assigned to the Rh^3+^ species.^[^ ^20^ ^]^ The detection of two additional peaks at binding energies of 308.1 and 312.8 eV signifies the existence of Rh species in a low oxidation state (Rh^δ+^, 0<δ<3).^[^ ^21^ ^]^ The low oxidation state Rh species may be induced by the oxygen vacancies adjacent to the Rh atoms. The high‐resolution O 1s spectra presented in Figure 2c can be deconvoluted into three distinct peaks, each corresponding to the binding energies of 530.0, 531.2, and 532.2 eV, respectively. These peaks are attributed to the lattice oxygen (O_L_), oxygen vacancy defects (O_V_), and adsorbed oxygen (O_A_) species.^[^ ^22^ ^]^ The peak area percentage of O_L_, O_V_, and O_A_ in TiO_2_ is 83.6%, 11.7%, and 4.74%, respectively. Meanwhile, the content O_V_ species in Rh_1_‐TiO_2_ catalyst exhibits a notable increase, with peak area percentage of 17.5%, which is significantly surpassing that of the TiO_2_ one (11.7%). This indicates that the incorporation of Rh single atoms into TiO_2_ results in a higher density of defect sites and oxygen vacancies. Figure 2d depicts the high‐resolution XPS spectra of Ti 2p, the Ti 2p core‐levels also split into 2p_3/2_ and 2p_1/2_ states due to the spin‐orbital coupling. The peak of Ti 2p_3/2_ for both Rh_1_‐TiO_2_ and TiO_2_ catalysts are situated at ≈458.7 eV, suggesting that the Ti species in these catalysts are primarily Ti^4+^, The peaks at 458.0 eV are correspond to the Ti^3+^ species, which is the coordinatively unsaturated Ti atoms near the oxygen vacancies.^[^ ^23^ ^]^ Ti^3+^/ Ti^4+^ in Rh_1_‐TiO_2_ is significantly higher than that of in TiO_2_, implying that the electronic structure of pristine TiO_2_ has been altered by the introduced rhodium atoms. XAFS was employed to further assess the valence state and probe the coordination environment of Rh and Ti in the Rh_1_‐TiO_2_ catalyst. The X‐ray Absorption Near Edge Structure (XANES) of the Rh K‐edge is presented in Figure 2e, The Rh K‐edge absorption edge in Rh_1_‐TiO_2_ lies between the Rh foil and Rh_2_O_3_ references, suggesting that the Rh atoms in the Rh_1_‐TiO_2_ catalyst are positively charged, with a valence state between Rh^0^ and Rh^3+^. The results are in good agreement with the XPS data. The Fourier‐transformed extended X‐ray absorption fine structure (EXAFS) spectra of the Rh K‐edge in the Rh_1_‐TiO_2_ catalyst exhibit a prominent peak at 1.5 Å, this peak can be attributed to Rh‐O coordination, as shown in Figure 2f. Compared to the EXAFS spectra of the Rh K‐edge in Rh_2_O_3_ and Rh foil, the Rh‐Rh coordination signal ≈2.4 Å is significantly weaken and it is almost negligible in that of Rh_1_‐TiO_2_ catalyst, confirming that Rh atoms are atomically dispersed on TiO_2_ matrix. The corresponding wavelet transform (WT) plot in Figure 2g highlights an intensity maximum at ≈6.7 Å^−1^, which is originated from Rh‐O scattering in Rh_1_‐TiO_2_. Despite the presence of similar Rh‐O scattering in Rh_2_O_3_, but the absence of Rh‐Rh scattering in the Rh_1_‐TiO_2_ catalyst, revealing the dispersion of isolated Rh atoms throughout the whole Rh_1_‐TiO_2_, further confirming the conclusion of that Rh atoms are atomically dispersed on Rh_1_‐TiO_2_ catalyst, rather than forming rhodium oxide clusters, which is consistent with the HAADF‐STEM results. The Ti K‐edge white line intensity of the Rh_1_‐TiO_2_ catalyst exhibited a marked increase compared to the Ti foil, while the absorption edge was slightly shifted toward lower energies compared to TiO_2_ (Figure S8, Supporting Information), suggesting that the Ti valence in the Rh_1_‐TiO_2_ catalyst is slightly lower than +4, with the presence of a minor fraction of Ti^3+^ species, which aligns with the XPS results.

**Figure 3:**
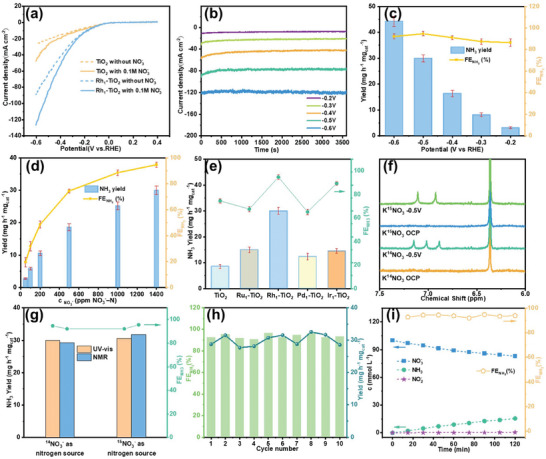
a) LSV curves of Rh_1_‐TiO_2_ and TiO_2_ electrodes in Ar‐saturated 0.1 m KOH in the absence and presence of 0.1 m KNO_3_. b) Chronoamperometry measurements of Rh_1_‐TiO_2_ electrodes in Ar‐saturated 0.1 m KOH +0.1 m KNO_3_ under varying applied potentials. c) NH_3_ yield rate and FE of Rh_1_‐TiO_2_ electrodes under various potentials during NO_3_RR. d) NH_3_ yield rate and FE of Rh_1_‐TiO_2_ electrodes for NO_3_RR at ‐0.5 V versus RHE in 0.1 m KOH containing 50, 100, 200, 500, and 1000 ppm NO_3_‐N, respectively. e) Comparison of NH_3_ yield rate and FE of TiO_2_, Ru_1_‐TiO_2_, Rh_1_‐TiO_2_ Pd_1_‐TiO_2,_ and Ir_1_‐TiO_2_ electrodes at ‐0.5 V versus RHE. f) ^1^H nuclear magnetic resonance (NMR) spectra of the electrolyte after NO_3_RR on Rh_1_‐TiO_2_ electrode at −0.5 V versus RHE with K^15^NO_3_ and K^14^NO_3_ as nitrogen sources. g) NH_3_ yield rate and FE of Rh_1_‐TiO_2_ electrodes at −0.5 V versus RHE by using ^15^NO_3_^−^ and ^14^NO_3_^−^ as the nitrogen sources, detected by colorimetric method (orange bars) and nuclear magnetic spectra methods (blue bars). h) Cyclic performance of Rh_1_‐TiO_2_ electrode at −0.5 V versus RHE over 10 cycles, with each cycle consisting of 1 h long‐term electrolysis. (i) Time‐dependent concentrations of NO_3_^−^, NO_2_^−^, and NH_3_ were monitored during the electrochemical NO_3_RR on the Rh_1_‐TiO_2_ electrode at the potential of −0.5 V versus RHE.

The NO_3_RR capabilities of the electrocatalysts were investigated in a standard H‐cell with a Nafion‐117 membrane separated the anode and cathode compartments. **Figure** **3**a presents the LSV curves of the electrodes in Ar‐saturated 0.1 m KOH as electrolyte, in the absence and presence of 0.1 m KNO_3_ as a nitrate source. The current densities of both the Rh_1_‐TiO_2_ and TiO_2_ electrodes significantly increased upon the addition of KNO_3_ electrolyte, indicating that both Rh_1_‐TiO_2_ and TiO_2_ are active toward electrocatalytic nitrate reduction. The Rh_1_‐TiO_2_ electrode exhibits a higher current density compared to the TiO_2_ electrode, revealing that the incorporation of single‐atom Rh enhances the electrocatalytic performance for nitrate reduction. The electrochemically active surface area (ECSA) of the electrocatalysts was calculated from the electrochemical double‐layer capacitance (Figure S12 and Table S2, Supporting Information).^[^ ^24^ ^]^ The calculated ECSA of the Rh_1_‐TiO_2_ catalyst is 35.2 m^2^ g^−1^, which is closed to that of the TiO_2_ (33.1 m^2^ g^−1^). The mass activity and specific activity were calculated from the NO_3_RR current normalized to the loading and ECSA of the catalyst on the working electrode, respectively. It should be noted HER and NO_3_RR are competing reactions at 0 to −0.6 V versus RHE, but the oxidation potential of NO_3_^−^ is higher than that of water, and the NO₃RR is more favorable than HER in the presence of NO_3_^−^ from the thermodynamic perspective. To quantify the ammonia yield rate and FE, chronoamperometric measurements of the catalysts were conducted at varying applied potentials. Figure 3b presents the chronoamperometry results obtained for the Rh_1_‐TiO_2_ electrode at varying applied potentials, the currents remain stable during the electrochemical nitrate reduction tests. After 1 h chronoamperometry test, the quantities of NH_3_ generated at different applied potentials were determined by using indophenol blue colorimetry on UV–vis spectrophotometer. The corresponding UV–vis chromogenic absorption spectra, depicted in Figure S13 (Supporting Information), indicate a gradual increase in absorbance as the applied potential was varied from −0.2 to −0.6 V versus RHE, this implies a higher yield rate of NH_3_ at the more negative potentials. Figure 3c illustrates the NH_3_ yield rate and FE of the Rh_1_‐TiO_2_ catalyst across varying potentials. As the potential decreases, the NH_3_ yield rate consistently increases. Conversely, the FE initially rises and subsequently declines, the highest FE is ≈94.7% at the potential of −0.5 V versus RHE, accompanied by an NH_3_ yield rate of 29.98 mg h^−1^ mg_cat_^−1^, which outperformed most of the Rh‐based, TiO_2_‐based, and single‐atom catalysts reported, as well as some catalysts evaluated in alkaline electrolytes (As summarized in Tables S3 and S4, Supporting Information), this underscores the remarkable activity and selectivity of our Rh_1_‐TiO_2_ catalyst. The contributions of other possible products to the faradaic current were also evaluated using gas chromatography equipped with thermal conductivity detector (TCD) and UV–vis spectrometry. Only H_2_ and NO_2_^−^ were detected (Figure S14, Supporting Information), and their FEs were calculated to be 4.1% and 2.1%, respectively. After taking the FE of NH_3_ into consideration, the total FE is very close to 100% as expected, further confirming its high selectivity toward NH_3_ production on the Rh_1_‐TiO_2_ catalyst. Figure 3d demonstrates that a decrease in the initial NO_3_^−^ concentration in the electrolyte leads to a reduction in both the NH_3_ yield rate and FE. This can be attributed to the presence of a competing reaction (HER) during the nitrate reduction process. Low NO_3_^−^ concentrations significantly enhance diffusion‐limited control during the reaction, leading to decreased selectivity toward nitrate reduction. The performance of the Rh_1_‐TiO_2_ toward NO_3_RR under neutral conditions (0.1 m PBS+0.1 m KNO_3_) was further investigated. Although the current density decreased in comparison to in alkaline electrolyte, the FE remains above 90% (Figure S15, Supporting Information). We also evaluated the electrochemical nitrate reduction performance of catalysts featuring varying rhodium loadings (Figure S16, Supporting Information). The results indicate that as the amount of rhodium single atoms decreases, the Rh_0.5_‐TiO_2_ electrode shows both current density and NH_3_ yield rate diminish, whereas FE remains exceptionally high, exceeding 90%. However, when the rhodium loading increases to ≈2%, the current density increases in LSV measurements, but the FE notably drops to ≈80%. This decrease of FE may stem from the presence of rhodium clusters in Rh_2_‐TiO_2_ catalyst, the rhodium clusters favors the hydrogen evolution reaction at the higher overpotemtials, thereby compromising the selectivity toward nitrate electrochemical reduction.^[^ ^11^, ^25^ ^]^ The results further confirm the high performance of Rh single atoms in NO_3_RR. Additionally, we assessed the electrochemical nitrate reduction performance of TiO_2_ and a range of noble metal single‐atom doped TiO_2_ catalysts, including Ru_1_‐TiO_2_, Pd_1_‐TiO_2_, and Ir_1_‐TiO_2_ (Figures S17 and S18, Supporting Information). Figure 3e summarizes the NH_3_ yield rate and FE of the catalysts at −0.5 V versus RHE. Notably, the Rh_1_‐TiO_2_ catalyst exhibits the highest NH_3_ yield rate and FE, significantly surpassing those of the other catalysts, indicating its superior catalytic activity and selectivity toward the electrochemical NO_3_RR. To definitively ascertain that the ammonia produced originated from electrochemical nitrate reduction on the Rh_1_‐TiO_2_ catalyst, as opposed to impurities, we conducted isotope labeling experiments using K^15^NO_3_ and K^14^NO_3_ as starting materials, followed by product characterization through ^1^H NMR analysis. As depicted in Figure 3f, the ^1^H NMR spectrum exhibits a characteristic splitting into triple peaks at 6.90, 7.03, and 7.16 ppm when utilizing ^14^NO_3_^−^ as the reactant. Conversely, double peaks are observed at the chemical shifts of 6.93 and 7.11 ppm when ^15^NO_3_^−^ is used.^[^ ^26^ ^]^ Additionally, when the chronoamperometric potential was set to the open circuit potential, no ammonia was detected in both ^14^NO_3_^−^ and ^15^NO_3_^−^ as the nitrogen source, indicating that the concentration of impurity ammonia in the reagent falls below the detection threshold. These results confirm that NO_3_^−^ in the electrolyte serves as the sole nitrogen source in the electrochemical synthesis of ammonia. Furthermore, we quantified the NH_3_ yield rate and FE by using ^1^H NMR spectroscopy and compared the results with those obtained from the colorimetric method (Figure S19, Supporting Information; Figure 3g). The results obtained using different methods are consistent. This further reinforces the conclusion that the ammonia obtained resulted from the electrochemical reduction of nitrate feedstock, rather than from contaminants. The durability of the Rh_1_‐TiO_2_ electrode for the electrochemical NO_3_RR was assessed through 10 consecutive cycles of electrolysis in an H‐cell electrolytic cell and conducted at −0.50 V versus RHE, which is the optimal potential for FE. As shown in Figure 3h, both the ammonia yield rate and FE remained stable, exceeding 28 mg h^−1^ mg_cat_^−1^ and at least 90.8%, respectively, indicating the remarkable stability of our Rh_1_‐TiO_2_ catalyst. Figure S20 (Supporting Information) presents the XRD, TEM, and HAADF−STEM results of Rh_1_‐TiO_2_ after 10 consecutive cycles in the stability test. Notably, no significant changes were observed in either the morphology or crystal structure, and no agglomeration of single atoms was detected following the stability test. During the stability test, the Rh_1_‐TiO_2_ electrode consistently demonstrated high catalytic performance while maintaining its structural integrity, thus confirming its remarkable durability. Figure 3i depicts the time‐dependent concentration changes of NO_3_^−^, NO_2_^−^, and NH_3_ during the electrochemical NO_3_RR of the Rh_1_‐TiO_2_ electrode at the potential of −0.5 V versus RHE. As the reaction progresses, the concentration of NO_3_^−^ gradually decreases and is converted into NH_3_, leading to an increase in the concentration of NH_3_ with a high FE (>90%), demonstrating the effective conversion of NO_3_^−^ to NH_3_. Additionally, the concentration of NO_2_^−^ is very low during the reaction. suggests the specific reduction of NO_3_^−^ and the formation of NH_3_ as the product.^[^ ^27^ ^]^

**Figure 4:**
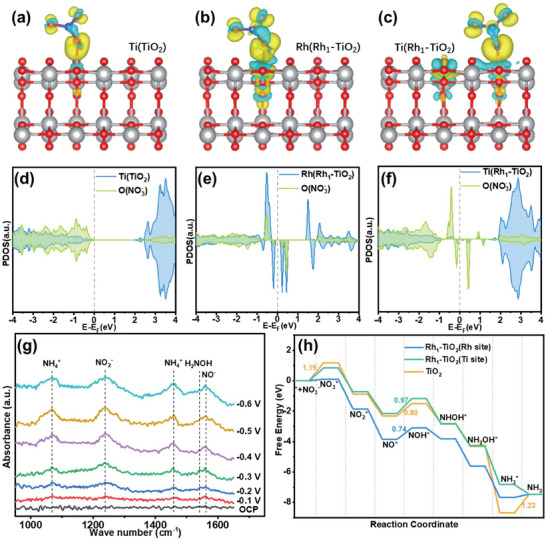
Charge density differences for NO_3_^−^ adsorbed on (101) surface of pristine anatase TiO_2_ a), Rh b), and Ti c) site of Rh_1_‐TiO_2_. (yellow and cyan represent electron accumulation and depletion, respectively, the isosurface is 0.003 e Å^−3^). The PDOS for NO_3_^−^ adsorbed on the anatase TiO_2_ d), Rh e), and Ti f) site of Rh_1_‐TiO_2_. g) In situ FTIR spectroscopy of Rh_1_‐TiO_2_ in 0.1 m KOH + 0.1 m NO_3_^−^ electrolyte at different potentials. h) Calculated free energy diagrams for different intermediates on anatase TiO_2_(orange line), Rh (blue line), and Ti (green line) site of Rh_1_‐TiO_2_ toward electrochemical nitrate reduction to NH_3_.

DFT calculations were performed to investigate the electrochemical NO_3_RR on TiO_2_ and Rh_1_‐TiO_2_ systems, the computational models are presented in Figure S21 (Supporting Information). The (101) surface of anatase TiO_2_ was chosen due to its stability.^[^ ^28^ ^]^ Given the differences in activity between Rh and Ti sites, both the Rh site and adjacent Ti sites on the (101) surface of Rh_1_‐TiO_2_ were considered. **Figure** **4**a–c illustrates the charge density differences upon NO_3_^−^ adsorption on pristine TiO_2_ and Rh_1_‐TiO_2_ at the Rh and Ti sites, respectively, highlighting substantial charge transfer interactions. Rh‐doping in TiO_2_ further enhances charge transfer, especially at the Rh and nearby Ti sites. This observation is further supported by the density of states analysis in Figure 4d–f, which reveals a pronounced d‐p orbital overlap near the Fermi level between Rh d‐states and NO_3_⁻ p‐states on the Rh_1_‐TiO_2_ surface. In contrast, the Ti‐NO_3_ interaction on pristine TiO_2_ exhibits weaker overlap. This suggests that the doping of a Rh single atom enhances the covalent bond strength between the active site and adsorbed NO_3_^−^, favoring its adsorption. Our analysis of the d‐band center further supports this observation. In pristine TiO_2_, the d‐band center of Ti is located at 1.74 eV, far from the Fermi level, indicating limited adsorption capability. In Rh_1_‐TiO_2_, the d‐band center of Ti slightly shifts to 1.86 eV, while the d‐band center of Rh is positioned at 0.12 eV, much closer to the Fermi level, indicating stronger electronic interaction and higher adsorption capability, making Rh a favorable active site for NO_3_RR.

We conducted in situ Fourier‐transform infrared (FTIR) spectroscopy to detect the intermediates adsorbed on the surface of Rh_1_‐TiO_2_ during the NO_3_RR in 0.1 m KOH + 0.1 m KNO_3_. As presents in Figure 4g, There is no obvious signal of adsorbed species on the catalyst surface under open circuit potential (OCP). With the increasement of applied potential, five signal peaks appeared gradually. The peaks located at 1238 and 1560 cm^−1^ are assigned to NO_2_^−^ and NO^−^ on the surface of catalyst, respectively.^[^ ^29^ ^]^ The two peaks located at 1068 and 1458 cm^−1^ are associated with the production of NH_4_^+^.^[^ ^30^ ^]^ The peak at 1538 cm^−1^ is correspond to the adsorbed NH_2_OH.^[^ ^31^ ^]^ On the basis of the above analysis, the potential reaction mechanism is summarized in Table S5 (Supporting Information). The free energy diagrams in Figure 4h illustrate the thermodynamics of the NO_3_RR pathways on TiO_2_ and at both the Ti and Rh sites on Rh_1_‐TiO_2_ surfaces. The results reveal that most reaction steps exhibit thermodynamic spontaneity, with the formation of NH_3_ being particularly favorable from the thermodynamic perspective. The rate‐limiting step for nitrate reduction on pristine TiO_2_ is the desorption of NH_3_ from the active site, requiring an energy input of 1.23 eV.^[^ ^32^ ^]^ In contrast, the rate‐limiting step for both Rh and Ti sites on Rh_1_‐TiO_2_ involves the formation of the NOH^*^ intermediate, which requiring energy inputs of 0.74 and 0.97 eV, respectively, significantly lower than the reaction energy barrier required for the rate‐determining step on pristine TiO_2_. Obviously, the lower reaction energy barrier required for the rate‐determining step on Rh_1_‐TiO_2_ surface reveals the faster electrochemical NO_3_RR and prove its high nitrate reduction activity. In summary, the high catalytic activity of Rh_1_‐TiO_2_ is primarily attributed to the high activity of the Rh site. Rh single‐atom doping provides a strong adsorption and activation site for NO_3_⁻ on TiO_2_, making Rh_1_‐TiO_2_ an excellent catalyst for NO_3_RR.

## 3 Conclusion

In conclusion, we have developed a facile spray‐drying method for large scale preparing Rh single‐atoms anchored on nanoporous TiO_2_ spheres, which serves as an efficient electrocatalyst for enhanced electrochemical reduction of NO_3_^−^ reduction to NH_3_. The atomically dispersed Rh atoms serve as highly active sites for NO_3_RR, while also optimizing the electronic structure of adjacent Ti sites and reducing the energy barrier for the rate‐limiting step. This results in a maximum FE of 94.7% and an NH_3_ yield rate of 29.98 mg h^−1^ mg_cat_^−1^ at −0.5 V versus RHE, significantly outperforming pristine TiO_2_ and other noble metal single‐atom catalysts (Ru, Pd, and Ir). This work highlights the spray‐drying based synthetic strategy for large scale synthesis of single‐atom noble metal materials. These single‐atom catalysts exhibit great potential for electrochemical NO_3_RR, offering novel insights into the NO_3_RR mechanism and serving as a roadmap for designing high‐performance NO_3_RR catalysts aimed at green ammonia synthesis.

## Conflict of Interest

The authors declare no conflict of interest.

## Supporting information



Supporting Information

## Data Availability

The data that support the findings of this study are available from the corresponding author upon reasonable request.
