# Supplementary material for: Isolated Rhodium Atoms Activate Porous TiO2 for Enhanced Electrocatalytic Conversion of Nitrate to Ammonia
Source: Adv Sci (Weinh). 2024 Nov 18;12(2):2411705. doi: 10.1002/advs.202411705 (PMC11727235; doi:10.1002/advs.202411705)
Supplement: Supplementary file 1 — Supporting Information [file ADVS-12-2411705-s001.docx]

Supporting Information

**Isolated Rhodium Atoms Activate Porous TiO_2_ for Enhanced Electrocatalytic Conversion of Nitrate to Ammonia**

Zhi Liang Zhao, Shaoxuan Yang, Shensong Wang, Zhe Zhang, Liang Zhao, Qi Wang*, Xinyi Zhang *

**Experiment section**

**Materials**

All of the reagents were used without further purification as received. Methyl methacrylate (> 99.5%, Contains 30ppm DMBP stabilizer), Sodium dodecyl sulfate (SDS, ≥ 99.0%), Potassium peroxydisulfate(99.5%), Titanium tetrachloride (TiCl_4_, 99.0%), Chloroauric Acid Hydrate (HAuCl_4_, 99%, Au: 50%), Chloroplatinic acid hexahydrate (H_2_PtCl_6_, 99%), Rhodium(III) Chloride Hydrate (RhCl_3_, 98%), Ruthenium (III) Chloride Hydrate (RuCl_3_, 97%), Dihydrogen Hexachloroiridate Hydrate (H_2_IrCl_6_, 98%) Ammonium Tetrachloropalladate(II) ((NH_4_)_2_PdCl_4_, Pd ≥ 36.5%), Potassium hydroxide (KOH, ≥ 99.0%), Sodium Nitroprusside(C_5_FeN_6_Na_2_O, 99%), Deuterium oxide(D_2_O, 99.9 atom % D), Maleic Acid(C_4_H_4_O_4_, 99%) Sulfuric Acid(H_2_SO_4_, 98%) Hydrochloric Acid (HCl, 37%) Phosphoric Acid (H_3_PO_4_, 85%) were provided from Shanghai Titan Technology Co., Ltd. Potassium nitrate-^15^N (K^15^NO_3_, 99 atom% ^15^N), Potassium nitrate (KNO_3_, 99%), Potassium nitrite (KNO_2_, AR, 97%), Ammonium chloride (NH_4_Cl, 99.5%), Salicylic acid (C_7_H_6_O_3_, ≥ 99%), sodium citrate (C_6_H_5_O_7_Na_3_, 98%), Sodium Hypochlorite Solution (NaClO, 1% (w/w)), N-(1-naphthyl) ethylenediamine dihydrochloride (C_12_H_14_N_2_·2HCl, > 98%) were provided from Sigma–Aldrich Chemical Reagent Co., Ltd.

**Material Characterizations**

The crystallographic structure of the catalysts was analyzed using X-ray diffraction (XRD) on a BRUKER D8 ADVANCE instrument. X-ray photoelectron spectroscopy (XPS) data for the samples were collected using a Thermo Scientific Escalab 250Xi spectrometer. SEM images were obtained by using Zeiss GeminiSEM 500 at accelerating voltage of 10 kV. The TEM, STEM and EDS experiments were conducted using a double Cs-corrected Titan Themis G2 microscope equipped with a dual Enfinium camera (Gatan) and acquired at an operating voltage of 300 kV. UV-Vis absorption spectrum was recorded on Agilent Cary 60. ^1^HNMR were tested on Bruker 400 MHz AVANCE III Nuclear Magnetic Resonance System. In-situ FTIR spectroscopy experiments were conducted on VERTEX 70v vacuum FT-IR spectrometer equipped with a mercury-cadmiumtelluride (MCT) detector cooled by liquid nitrogen. The gas products are detection by gas chromatography (Agilent 8890) with a thermal conductivity detector.

**Synthesis of PMMA spheres:**

Polymethylmethacrylate (PMMA) spheres were synthesized via emulsion polymerization, following a previously reported protocol. ^1^. Briefly, 250 mL of water was refluxed at 40 °C for 30 minutes under a nitrogen flow to remove dissolved oxygen. Subsequently, 17.8 g of MMA and 5.0 mg of SDS were added to the stirring water. Thereafter, the temperature was raised to 90°C and maintained for 30 minutes. A solution of 150 mg of potassium peroxydisulfate in 4 mL of water was injected into the aforementioned emulsion, and the reaction mixture was maintained at 90°C for an additional 3 hours under a nitrogen flow. The suspension of PMMA spheres was collected by centrifugation and washed with water twice. Finally, the PMMA spheres were redispersed in 200 mL of water.

**Synthesis of porous TiO_2_** **microspheres and Rh single-atom noble metal on porous TiO_2_ microspheres:**

In a typical synthesis, the single-atom Rh on porous TiO_2_ microspheres (Rh_1_-TiO_2_) is prepared by a facile spray-drying process followed by thermal annealing. 1.5mL of TiCl_4_ was added dropwise to 10 mL of ethanol under stirring conditions, and subsequently add 150 mL of deionized water. After stirring another 10min, 2mL solution contained 35mg of RhCl_3_ and 20 mL of PMMA dispersed solution was added and maintain stirring until a uniform mixture is achieved. The mixed solution was spray-dried at 160 °C and then the resulting precursor product was placed in a muffle furnace and heated to 400°C in air with a heating rate of 2°C/min and maintained at 400°C for 3 hours. The final product obtained is single-atom Rh on porous TiO_2_ microspheres. The loading of rhodium within the materials can be adjusted by varying the number of Rh precursors. The preparation procedure of Rh_0.5_-TiO_2_ and Rh_2_-TiO_2_ is similar to that of Rh_1_-TiO_2_, except that addition amount of RhCl_3_ was 17.5 mg and 70 mg, respectively.

For the synthesis of single-atom Ru on porous TiO_2_ microspheres (Ru_1_-TiO_2_), single-atom Pd on porous TiO_2_ microspheres (Pd_1_-TiO_2_), single-atom Ir on porous TiO_2_ microspheres (Ir_1_-TiO_2_), the preparation process is similar to that of Rh_1_-TiO_2_, except that the rhodium salt is replaced with RuCl_3_, (NH_4_)_2_PdCl_4_ and H_2_IrCl_6_, respectively. We also prepared the Pt and Au doped porous TiO_2_, but XRD results show the Pt and Au are not single atom dispersed on TiO_2_, but are particles (**Figure S7**).

Electrochemical experiments were tests were done on an electrochemical workstation (Autolab 302N) and carried out using a H–type cell. A Nafion-117 membranes treated with 5% H_2_O_2_, 5% H_2_SO_4_, and H_2_O at 80℃ for 1 h, respectively, were used to separate the anode and cathode chambers. A Pt mesh (1×1cm^2^) was used as the counter electrode and a Ag/AgCl served as the reference electrode. All potentials were referenced to a reversible hydrogen electrode (RHE) by *E* (V vs. RHE) = *E* (V vs. Ag/AgCl) + 0.198 V + 0.059 × pH. The catalyst ink was prepared by dissolving 2 mg of the catalyst in 0.75 mL of ethanol, 0.23 mL water and 20 µL of Nafion ionomer solution (5 wt%). After ultrasonic became uniform dispersion, the catalyst ink was dropped onto a carbon cloth (0.5×0.5 cm^2^) to attain a catalyst loading of ~0.2 mg cm^–2^. During the electrocatalysis tests, the anode and cathode chamber was filled with 40 mL of 0.1 M KOH+ 0.1 M KNO_3_. Potentiostatic tests were conducted during the NO_3_RR at different potentials under ambient conditions after 30 min of purging with Ar (99.999%). Chronoamperometry tests were conducted for 3600s at certain potentials to evaluate the catalytic performance of NO_3_^-^ electroreduce to NH_3_. After electroreduction, the concentration of NH_3_ in the electrolyte was measured by the indophenol blue method on the UV-visible spectrometer (Agilent Cary 60).

**Product detection:**

After dilution to the proper concentration to match the range of the calibration curve, the electrolyte's ion concentration is measured using an ultraviolet-visible (UV-Vis) spectrophotometer.

**Determination of NH_3_**

The generated NH_3_ in cathode chamber electrolyte was determined by the indophenol blue method through ultraviolet absorption spectroscopy (Li, Jin, Fang, & Yu, 2021). Before the UV test, the cathode electrolyte was diluted to the detection range. Then, 2 ml of diluted electrolyte solution, 2 ml of 1.0 M KOH solution containing 5 wt% salicylic acid and 5 wt.% sodium citrate, 1 mL of 0.05 M NaClO solution and 0.2 mL of 1wt.% C_5_FeN_6_Na_2_O solution was added into a brown glass bottle and placed in dark environment. After 2 hours, the solution was measured by UV-vis spectrophotometry and using the absorbance at 655 nm wavelength. The standard concentration–absorbance curve was calibrated by a series of concentrations of the standard NH_4_Cl solution (**Figure S9**).

In addition, isotope-labelled nitrate reduction experiment was also conducted to verify the source of ammonia produced in this work. 0.1 M KOH and 0.1 M of K^15^NO_3_ or K^14^NO_3_ was added to the cathode chamber of H-type electrolytic cell. After electrolysis, the pH of the electrolyte in cathode chamber was adjusted to 4 by using of 1 M H_2_SO_4_. then 0.9 mL of the electrolyte, 80 μL of 25 gg mL^-1^ maleic acid and 100 uL of D_2_O were mixed in the NMR tube for ^1^H NMR measurements.

**Determination of NO_2_^–^**

The concentrations NO_2_^–^ in electrolyte was determined by the technique of ultraviolet (UV) absorption spectroscopy. The coloring solution was prepared by dissolving 0.2 g of N–(1–naphthyl) ethylenediamine dihydrochloride, 4 g of p-aminobenzene sulfonamide and 10 mL of H_3_PO_4_ into 100 of deionized water. For determine th concentrations NO_2_^–^, 5 mL of diluted electrolyte solution was mixed with 0.1 mL coloring solution in a brown glass vial and placed in a dark environment at room temperature. After 15 min, the mixture was subjected to UV–vis measurements and the adsorption peak appeared at 540 nm. The standard concentration–absorbance curve was calibrated by a series of concentrations of the standard KNO_2_ solution (**Figure S10**).

**Determination of NO_3_^–^**

The concentrations NO_3_^–^ in electrolyte was determined by the technique of ultraviolet (UV) absorption spectroscopy. 5 mL of diluted electrolyte solution was mixed with 0.1 mL of 1 M HCl solution and 10 uL of 0.8 wt.% sulfamic acid solution in a brown glass vial and placed in a dark environment at room temperature. After 15 min, the mixture was subjected to UV–vis measurements between the wavelengths of 200 nm and 300 nm. The final NO_3_^–^ absorbance was calculated using the formula A = A_220nm_ - A_275nm_. The standard concentration–absorbance curve was calibrated by a series of concentrations of the standard KNO_3_ solution (**Figure S11**).

**Analysis and calculation of products.** For the nitrate reduction reaction, the conversion rate (C (NO_3_^–^) %) of NO_3_^–^ can be obtained based on equation (1):

C(NO_3_^–^) %=[C_0_(NO_3_^–^)-C_t_ (NO_3_^–^)]/C_0_(NO_3_^–^) ×100% (1)

The selectivity of the NH_3_ and NO_2_^–^ can be calculated according to equation (2) and (3), respectively:

S(NH_3_) %= C_t_ (NH_3_)/ [C_0_ (NO_3_^–^)- C_t_ (NO_3_^–^)] ×100% (2)

S(NO_2_^–^) %= C_t_ (NO_2_^–^)/ [C_0_ (NO_3_^–^)- C_t_ (NO_3_^–^)] ×100% (3)

The Faradaic efficiency (FE) of NH_3_ can be calculated according to equation (4)

FE(NH_3_) = {[n×C_t_ (NH_3_) ×V×F]/1000×M×Q} ×100% (4)

the yield rate of NH_3_ produced from NO_3_^–^ can be calculated according to equation (5)

NH_3_ yield= {[C_t_ (NH_3_) ×V]/(M×t×S)} ×100% (5)

Where C_0_ (NO_3_^–^) is the initial concentration of NO_3_^–^ in the cathode chamber, C_t_ (NO_3_^–^ is the concentration of NO_3_^–^ after electrolysis, C_t_ (NH_3_) and C_t_ (NO_2_^–^) is the concentration of NH_3_ and NO_2_^–^ in the cathode chamber after electrolysis , V is the volume of the electrolyte in the cathode chamber, t is the electrolysis time, S is the area of working electrode (0.25 cm^2^), F is the Faradaic constant (96485 C mol^-1^) and Q is the total charge passing the working electrode.

**DFT calculation**

We utilized the Vienna Ab Initio Simulation Package (VASP) ^2^ ^3^ to conduct density functional theory (DFT) calculations employing the generalized gradient approximation (GGA) framework with the PBE ^4^ exchange-correlation functional. We employed projected augmented wave (PAW) potentials ^5^ ^6^ to represent the ionic cores, while accounting for valence electrons utilizing a plane wave basis set with a kinetic energy cutoff set at 450 eV. The Gaussian smearing method, with a width of 0.05 eV, was utilized to allow for partial occupancies of the Kohn-Sham orbitals. Self-consistency of the electronic energy was achieved when the energy variation fell below 10^−5^ eV. Convergence of the geometry optimization was deemed achieved when the force change diminished below 0.05 eV/Å. ^7^

We constructed a TiO_2_ (101) surface and introduced a vacuum layer of 15 Å depth along the z-direction to isolate the surface slab from its periodic images. The bottom two stoichiometric layers were kept fixed, whereas the remaining layers were allowed to relax.

The free energy of a gas phase molecule or an adsorbate on a surface was estimated using the equation G = E + ZPE - TS, wherein E represents the total energy, ZPE is the zero-point energy, T is the temperature (set at 298.15 K), and S signifies the entropy.


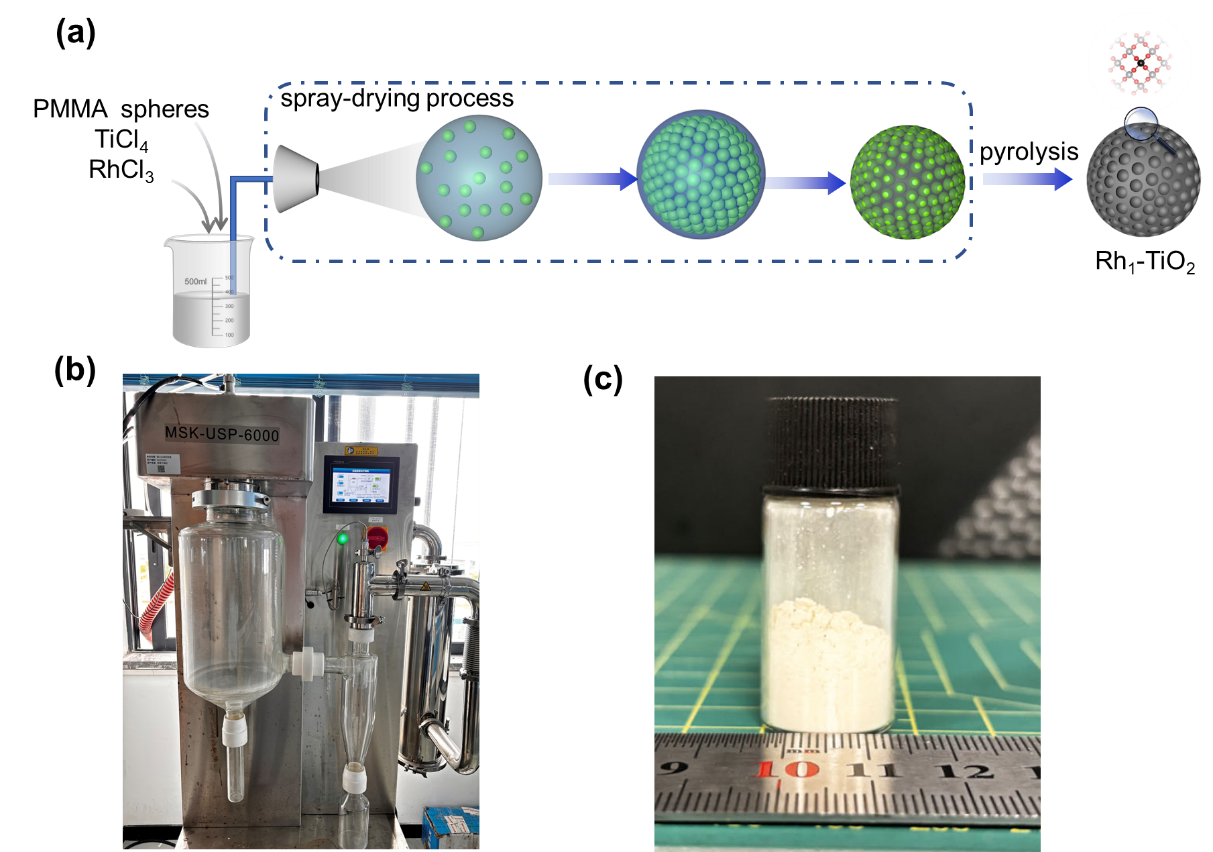


**Figure S1**. (a) Depicts the preparation of Rh_1_-TiO_2_ through spray-drying and pyrolysis process. Photograph of the spray dryer (b) used in our lab and the as-prepared Rh_1_-TiO_2_ (c).


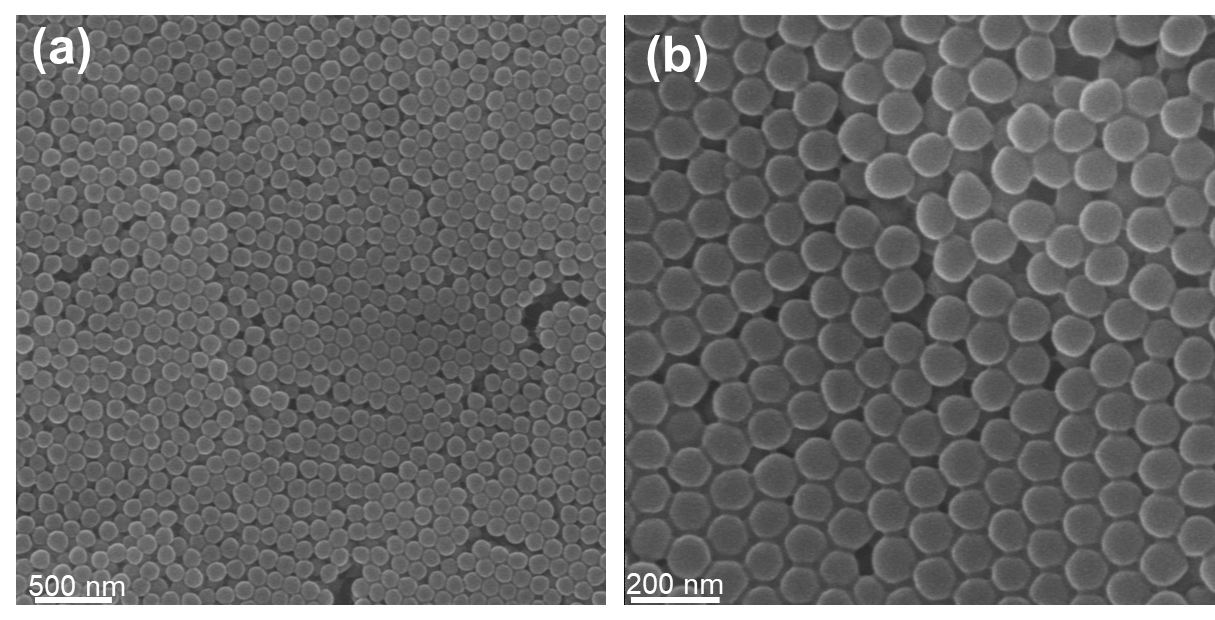


**Figure S2**. SEM images of PMMA microspheres.


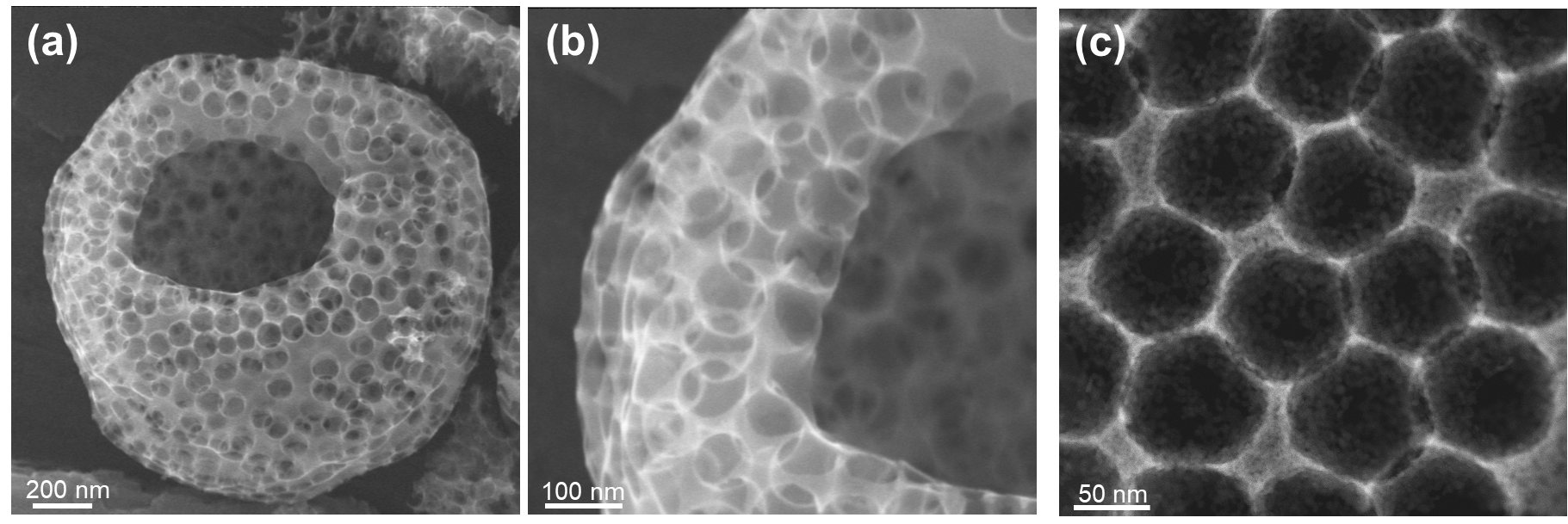


**Figure S3**. SEM images of a broken hollow spheres.


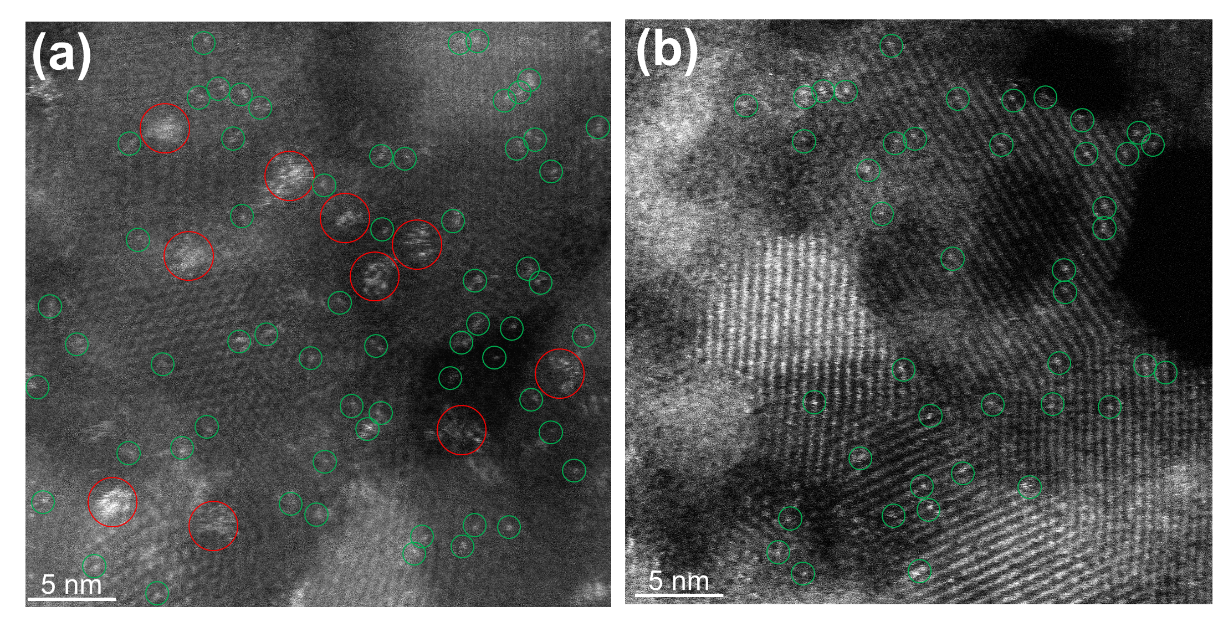


**Figure S4**. HAADF-STEM image of the catalysts with different rhodium loading of 2 at. % (a, Rh_2_-TiO_2_) and 0.5at. % (b, Rh_0.5_-TiO_2_), in which Rh single atoms are highlight with green circles and Rh clusters with are highlight with red circles.


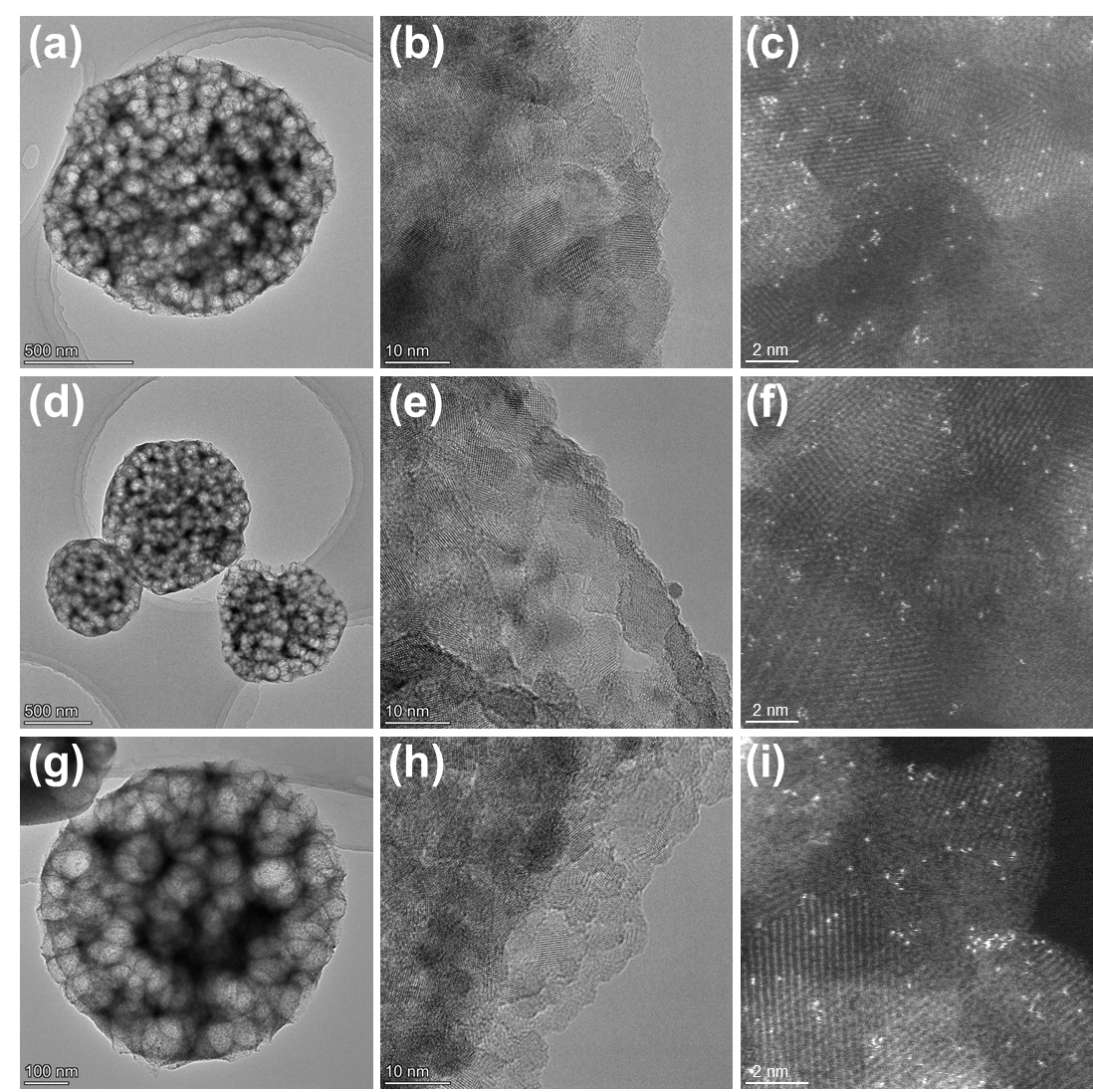


**Figure S5**. TEM images of the Ru_1_-TiO_2_ (a-c), Pd_1_-TiO_2_ (d-f) and Ir_1_-TiO_2_ (g-i) single-atom catalysts

**Figure S6**. XRD patterns of TiO_2_, Ru_1_-TiO_2_, Rh_1_-TiO_2_, Pd_1_-TiO_2_ and Ir_1_-TiO_2_, including reference peak positions of anatase TiO_2_ (JCPDS file No.21-1272).


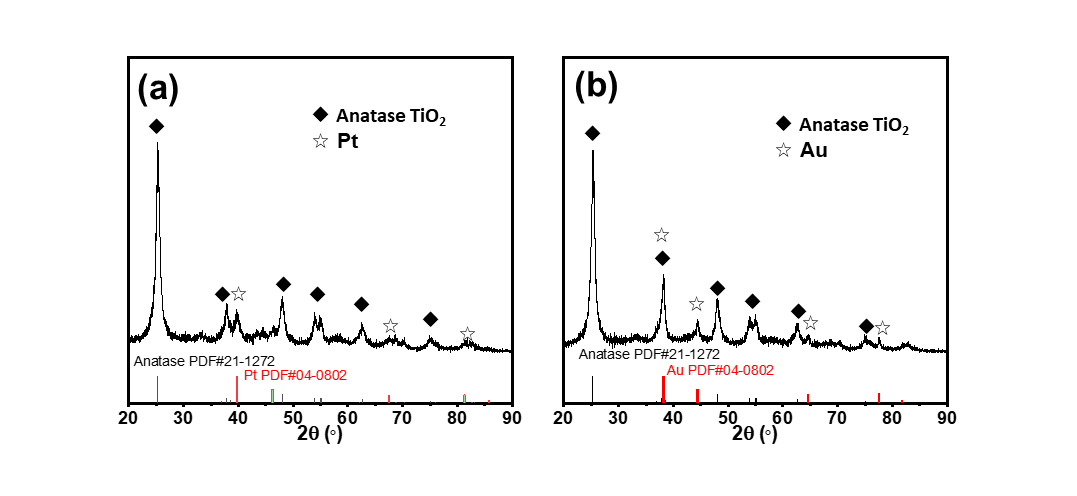


**Figure S7**. XRD patterns of the as-prepared samples with Pt and Au precursor. Since the oxidation potentials of Pt and Au precursor, the Au and Pt would be reduced by the reduced by gases produced during the thermal decomposition of organic matter during heat treatment, ultimately resulting in agglomeration of metallic platinum and gold atoms and resulting nanoparticles


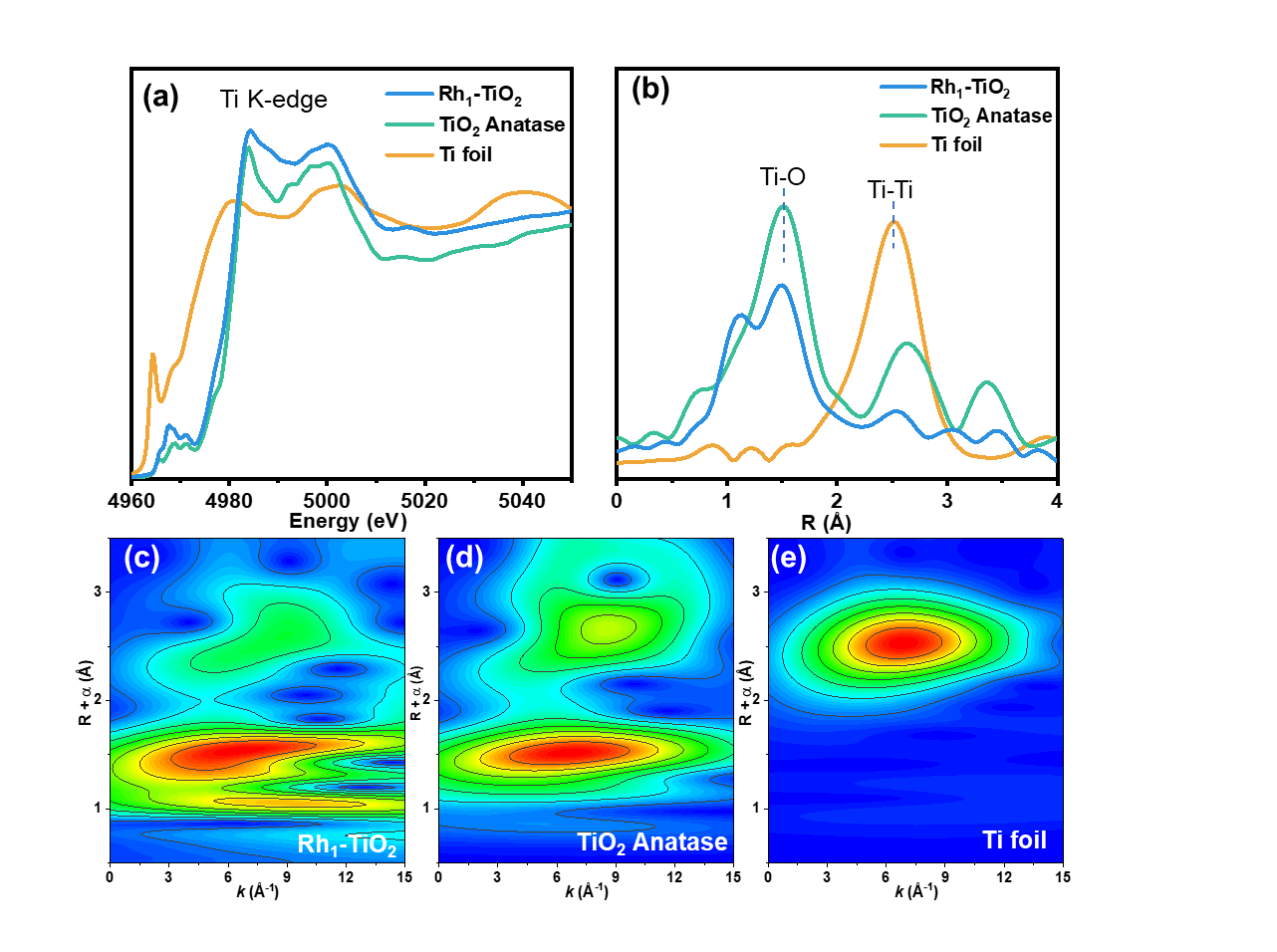


**Figure S8**. (a) XANES spectra of Rh_1_-TiO_2_ (Blue line), Ti foil (Orange line) and anatase TiO_2_ (Green line) at the Ti K-edge and the corrspording FT-EXAFS spectra (b). The k^3^-weighted WT-EXAFS spectra of (c) Rh_1_-TiO_2_, Ti foil (d) and anatase TiO_2_ (e).


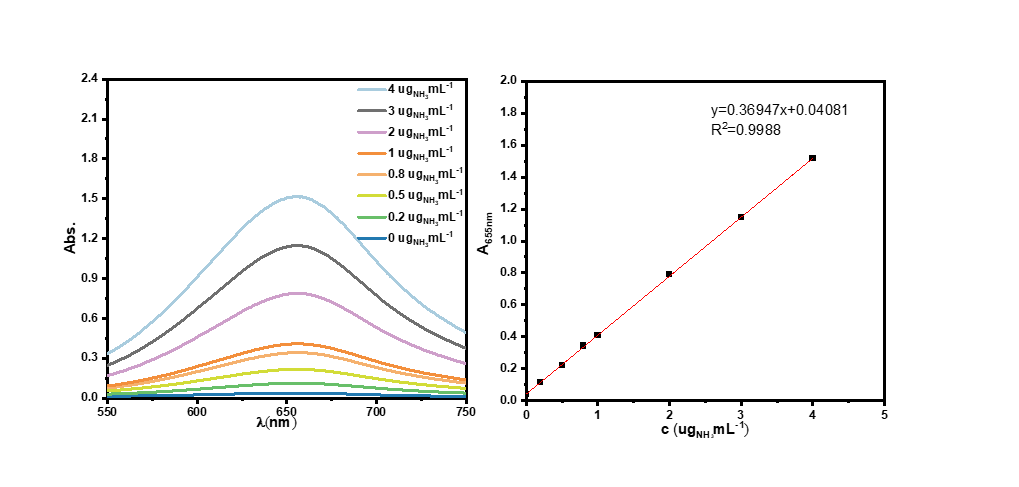


**Figure S9**. (a) UV-vis absorption spectroscopy for various concentrations of NH_4_Cl. (b) The standard NH_4_^+^ concentration–absorbance at 655nm (A_655nm_) curve.


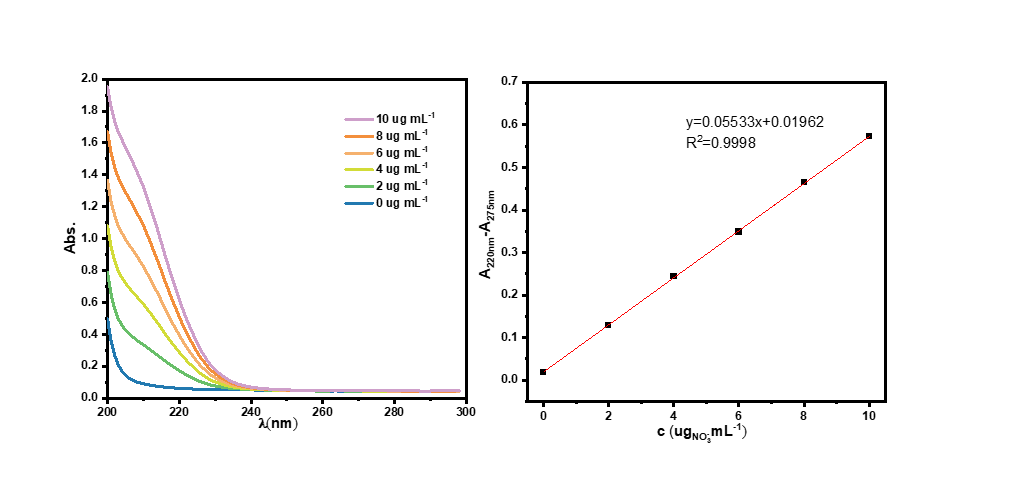


**Figure S10**. (a) UV-vis absorption spectroscopy for various concentrations of KNO_3_. (b)

The standard NO_3_^–^ concentration–absorbance (A_220nm_ - A_275nm_) curve.


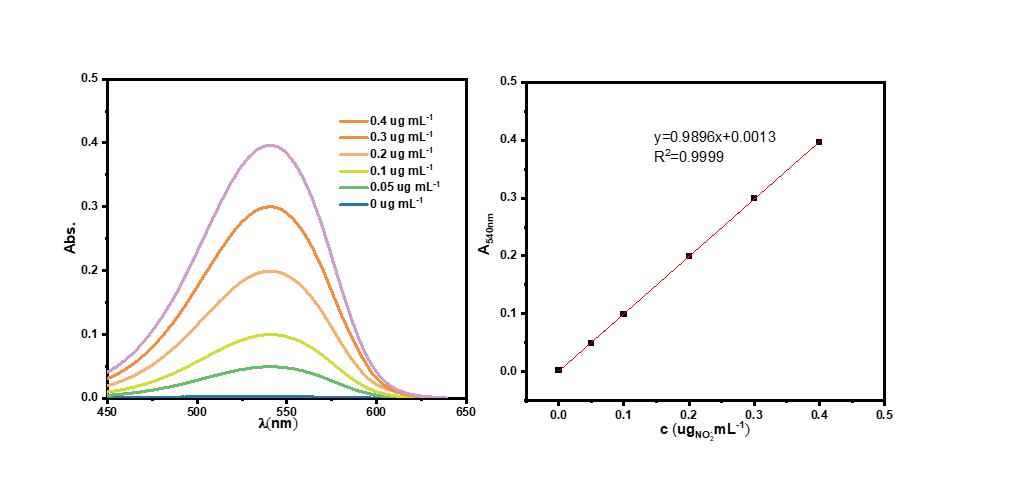


**Figure S11**. (a) UV-vis absorption spectroscopy for various concentrations of KNO_2_. (b) The standard NO_2_^–^ concentration–absorbance at 540nm (A_540nm_) curve.


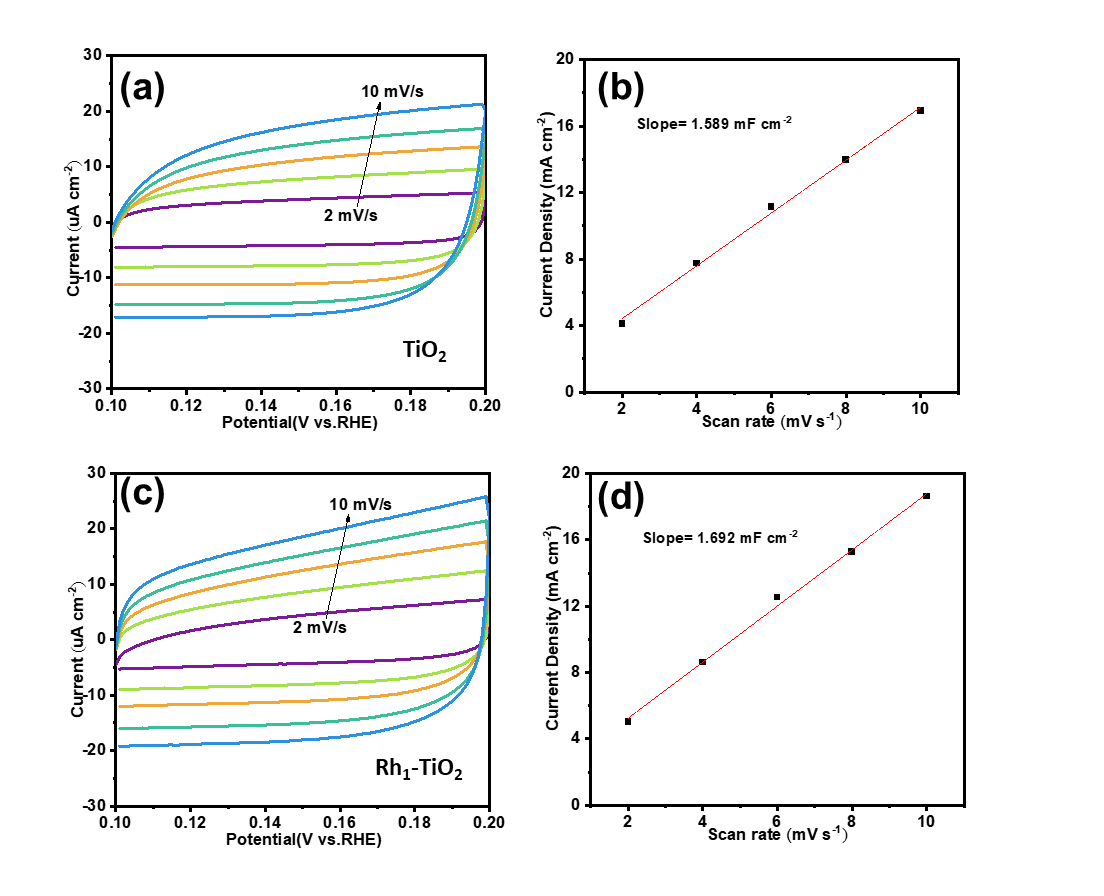


**Figure S12**. CV curves of (a) TiO_2_ and (c) Rh_1_-TiO_2_ in 0.1M KOH at different scan rates. (b) and (d) are corresponding plots of the current density at 0.15 V *vs.* the scan rate.


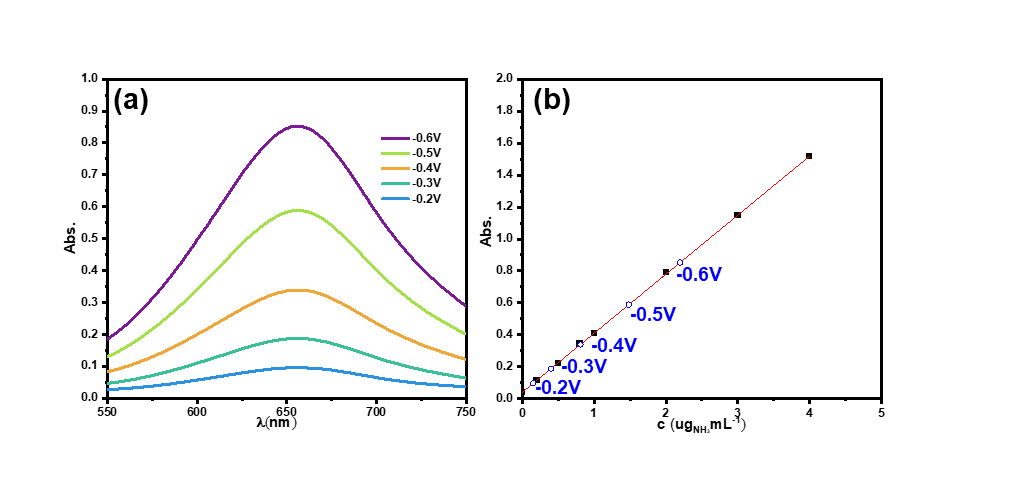


**Figure S13**. (a) UV-vis absorption spectroscopy of Rh_1_-TiO_2_ at varying applied potentials. (b) The standard NH_4_^+^ concentration–absorbance at 655nm (A_655nm_) curve. The blue circle represents the concentration of NH_4_^+^ after NO_3_RR at the applied potentials.


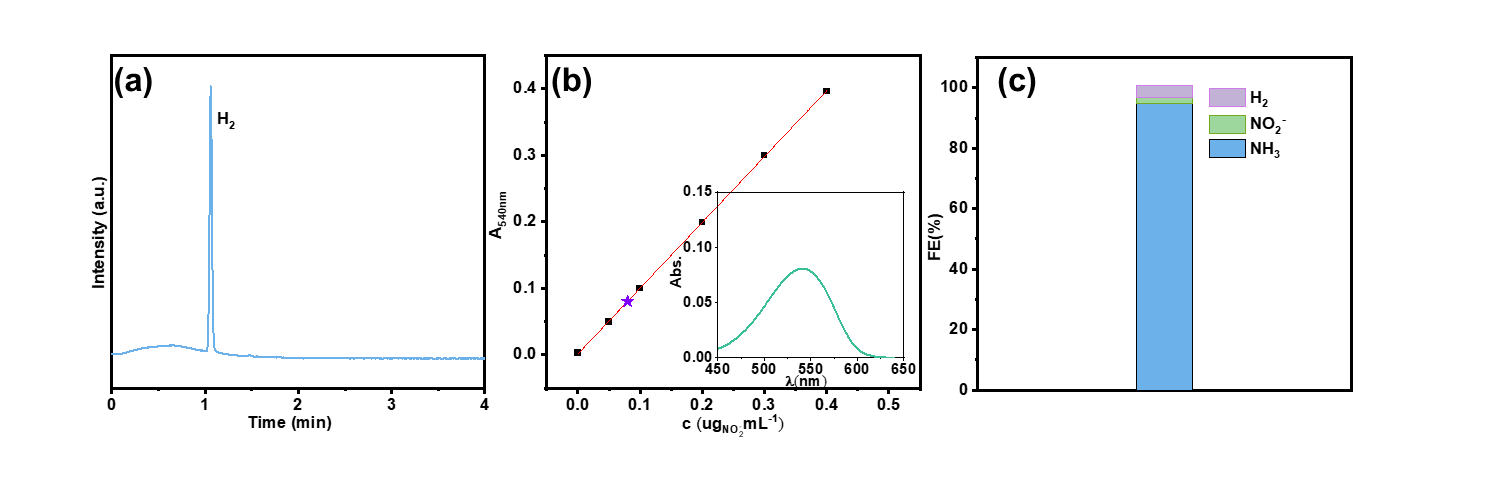


**Figure S14**. (a) The detection of gas products during NO_3_RR. Representative GC of gas products obtained on Rh_1_-TiO_2_ electrode at -0.5 V vs. RHE. (b) The standard NO_2_^-^ concentration–absorbance at 540 nm (A_540nm_) curve. The purple star represents the concentration of NO_2_^-^ after NO_3_RR on Rh_1_-TiO_2_ electrode at -0.5 V vs. RHE and the inset shows the cospording UV-vis absorption spectroscopy. (c) The FE of H_2_, NO_2_^–^ and NH_3_ during NO_3_RR on Rh_1_-TiO_2_ electrode at -0.5 V vs. RHE


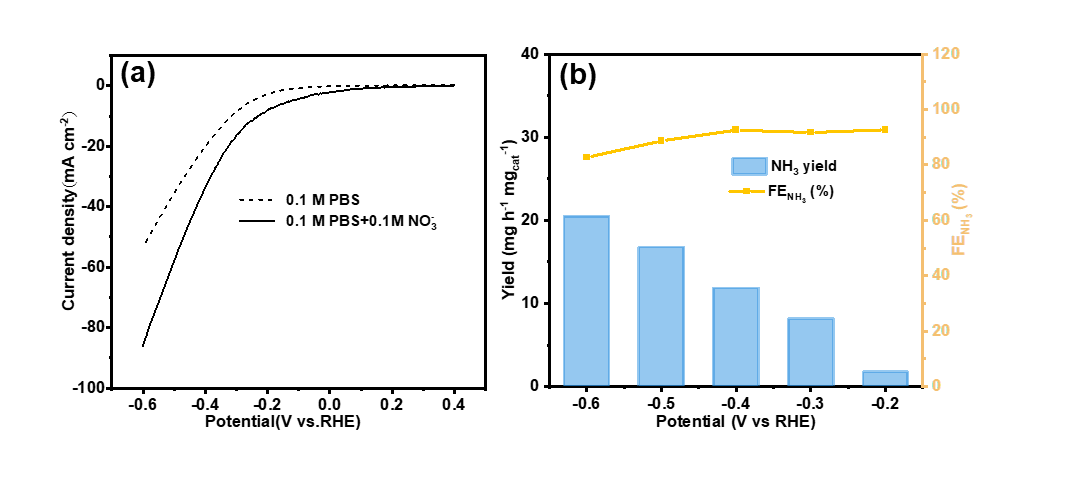


**Figure S15**. (a) LSV curves of Rh_1_-TiO_2_ in Ar-saturated 0.1 M PBS (pH=7) in the absence and presence of 0.1 M KNO_3_. (b) NH_3_ yield rate and FE of Rh_1_-TiO_2_ electrodes under various potentials during NO_3_RR in neutral electrolyte.


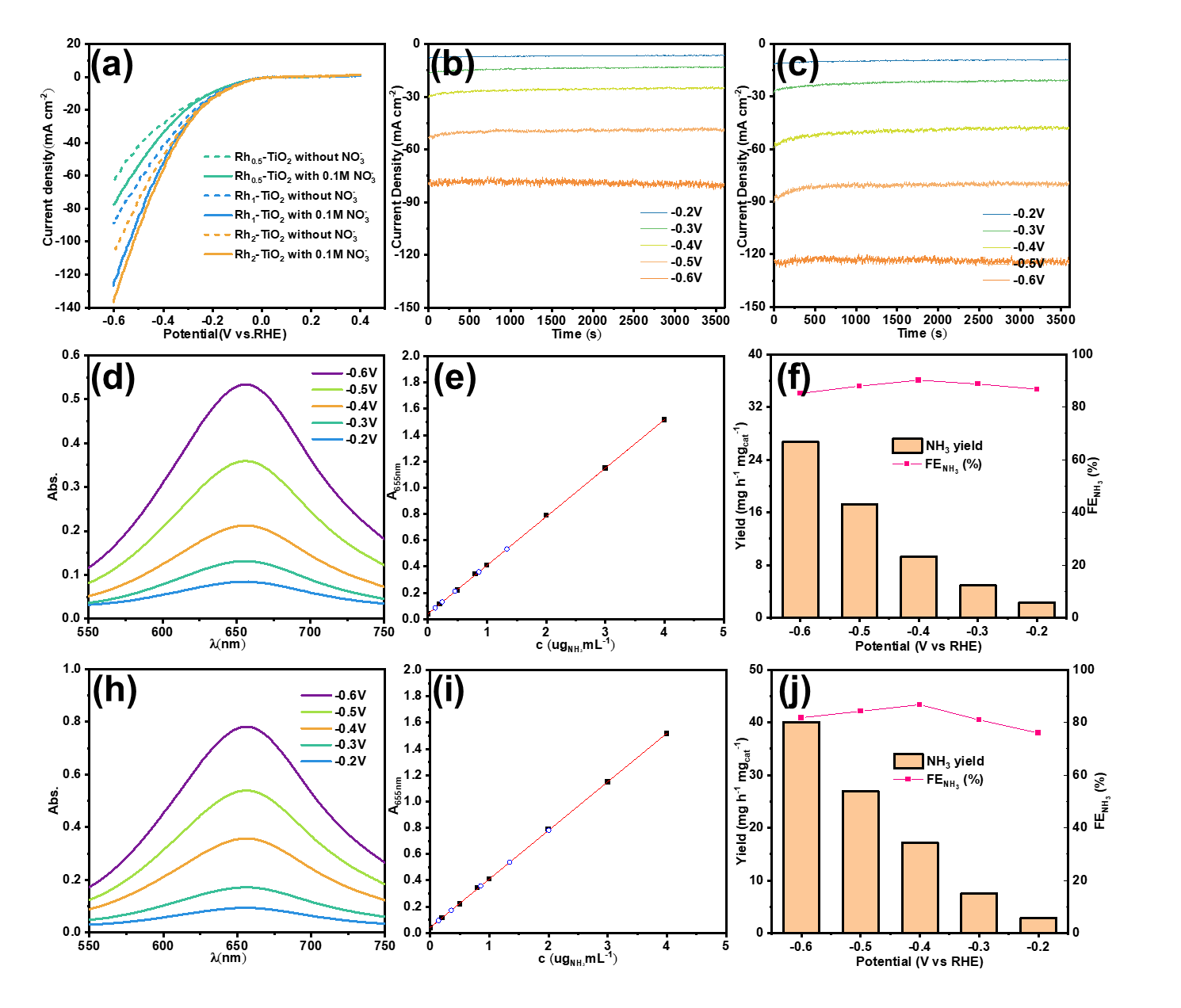


**Figure S16**. (a) LSV curves of the catalysts with different rhodium loadings (Rh_0.5_-TiO_2_, Rh_1_-TiO_2_ and Rh_2_-TiO_2_) electrodes in Ar-saturated 0.1 M KOH in the absence and presence of 0.1 M KNO_3_. Chronoamperometry measurements of Rh_0.5_-TiO_2_ (b) and Rh_2_-TiO_2_ (c) electrodes i 0.1 M KOH +0.1 M KNO_3_ under varying applied potentials. (d) UV-vis absorption spectroscopy, (e) the calculated concentration of ammonia, (f) ammonia yield ration and FE of Rh_0.5_-TiO_2_ at varying applied potentials. (h) UV-vis absorption spectroscopy, (i) the calculated concentration of ammonia, (j) ammonia yield ration and FE of Rh_0.5_-TiO_2_ at varying applied potentials. The blue circle represents the concentration of NH_4_^+^ after NO_3_RR at the applied potentials.


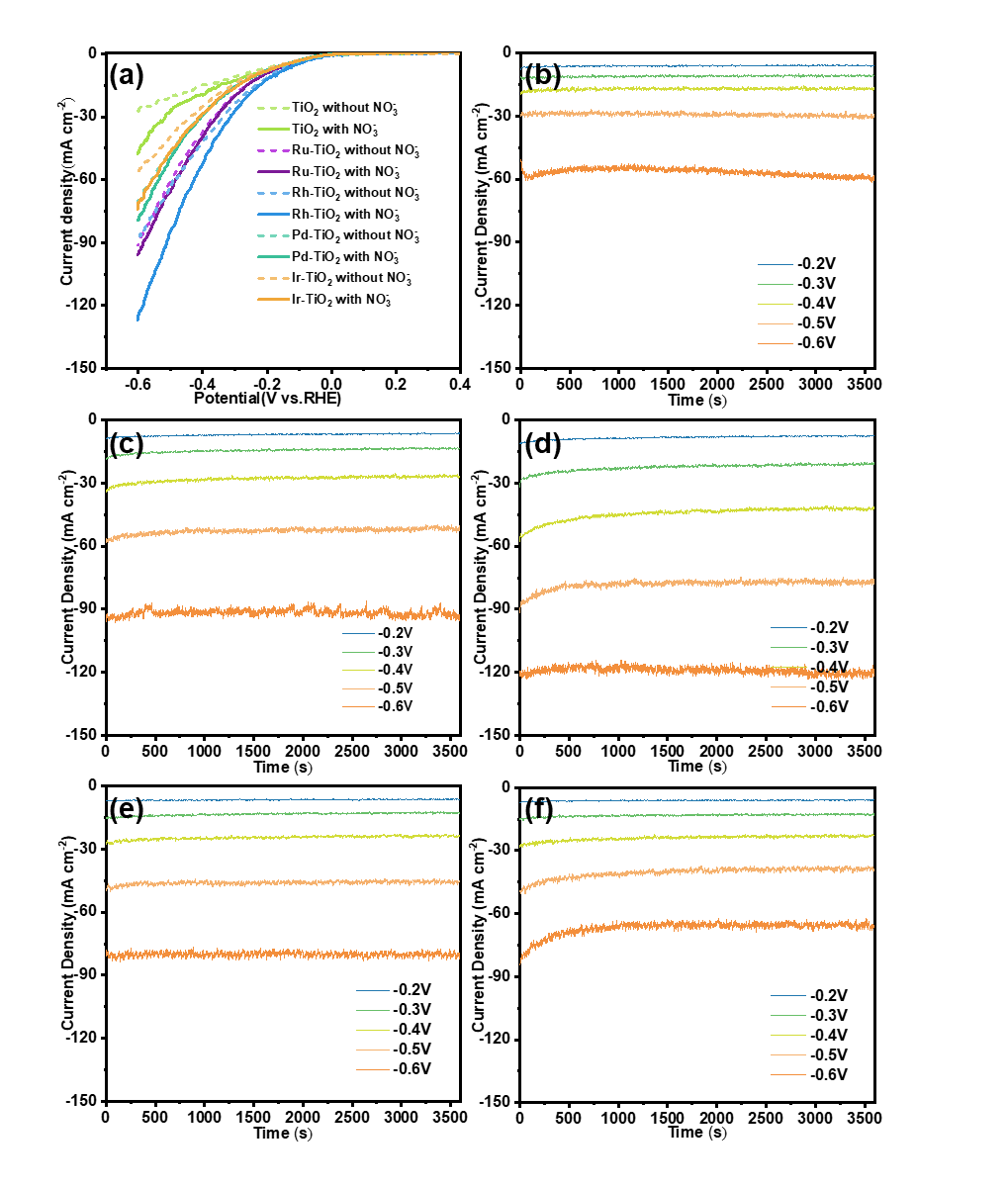


**Figure S17**. (a) LSV curves of varying single atom catalysts and TiO_2_ electrodes in Ar-saturated 0.1 M KOH in the absence and presence of 0.1 M KNO_3_ and corresponding chronoamperometry measurements of (b) TiO_2_, (c) Ru_1_-TiO_2_, (d) Rh_1_-TiO_2_, (d) Pd_1_-TiO_2_ and (e) Ir_1_-TiO_2_ catalysts under the applied potentials.


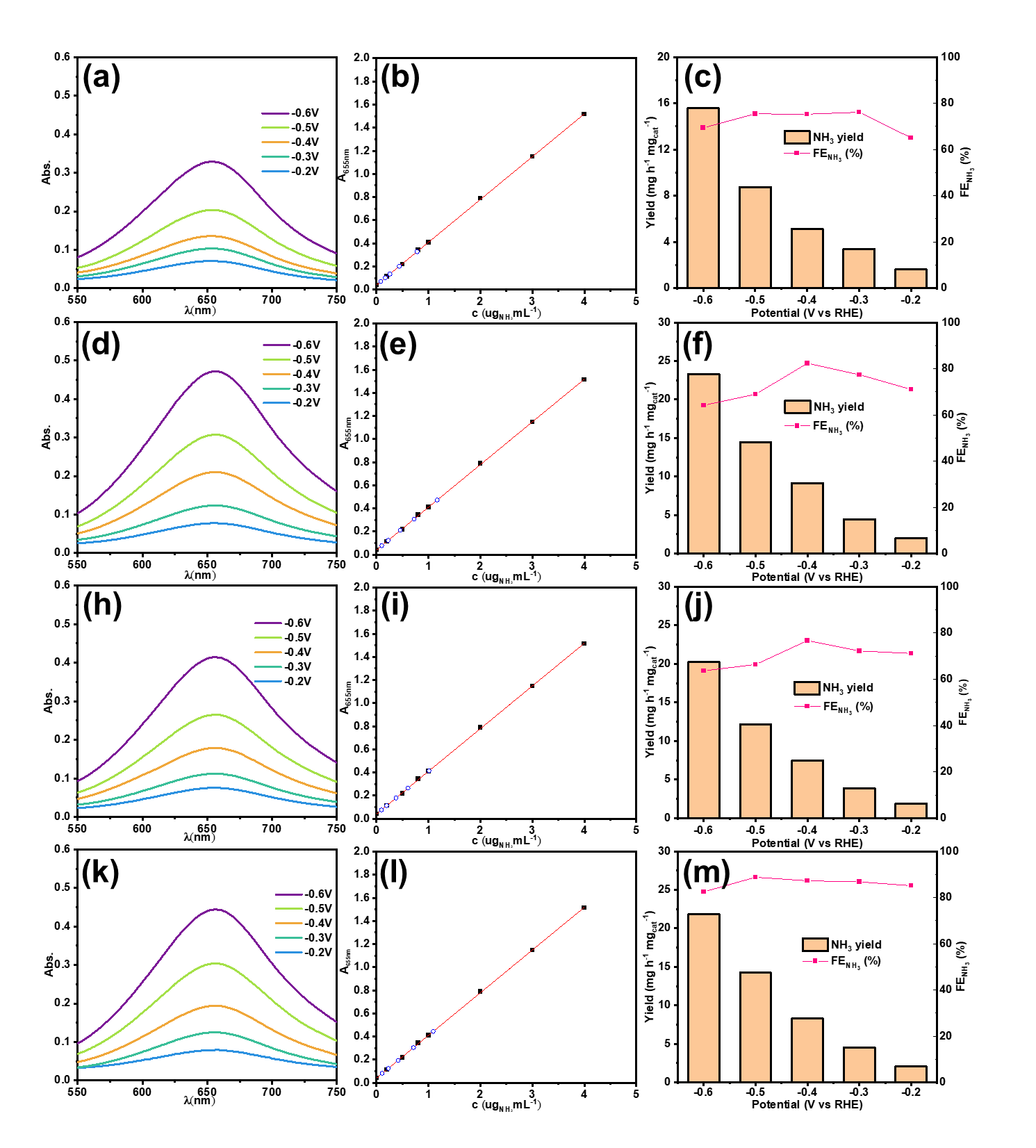


**Figure S18**. UV-vis absorption spectroscopy of TiO_2_ (a), Ru_1_-TiO_2_ (d), Pd_1_-TiO_2_ (h) and Ir_1_-TiO_2_ (k) at varying applied potentials. The calculated concentration of ammonia, ammonia yield ration and FE of TiO_2_ (b and c), Ru_1_-TiO_2_ (e and f), Pd_1_-TiO_2_ (i and j) and Ir_1_-TiO_2_ (l and m) at the applied potentials. The blue circle represents the concentration of NH_4_^+^ after NO_3_RR at the applied potentials.


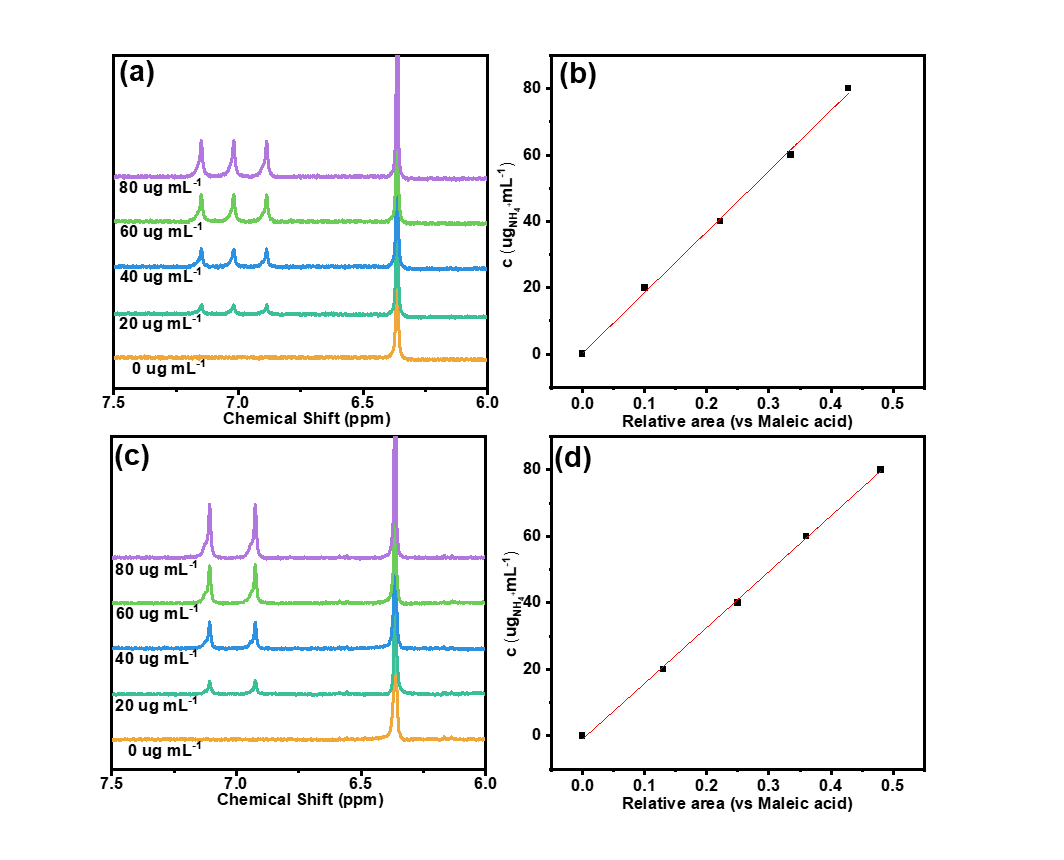


**Figure S19**. (a) ^1^HNMR for various concentrations of ^14^NH_4_Cl and (b) the standard curve of integral area (NH_4_^+^/C_4_H_4_O_4_) as a function of ^14^NH_4_^+^concentration. (c) ^1^HNMR for various concentrations of ^15^NH_4_Cl and (d) the standard curve of integral area (NH_4_^+^/C_4_H_4_O_4_) as a function of ^15^NH_4_^+^concentration.


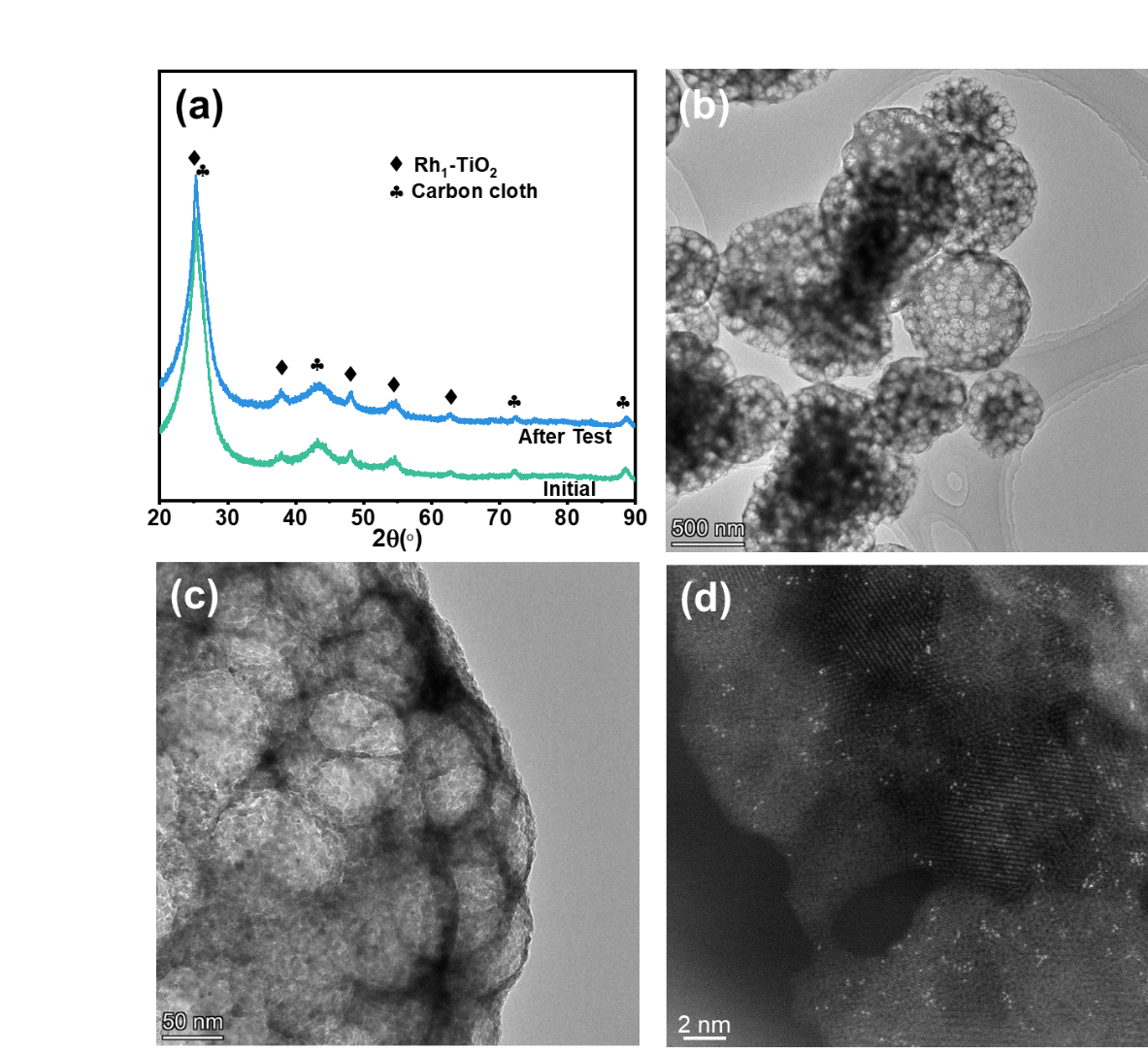


**Figure S20**. (a) XRD of the carbon cloth supported Rh_1_-TiO_2_ catalyst as the working electrode before and after the stability test. (b,c) TEM and (d and inset ) HAADF−STEM images of the Rh_1_-TiO_2_ catalyst after the stability test.


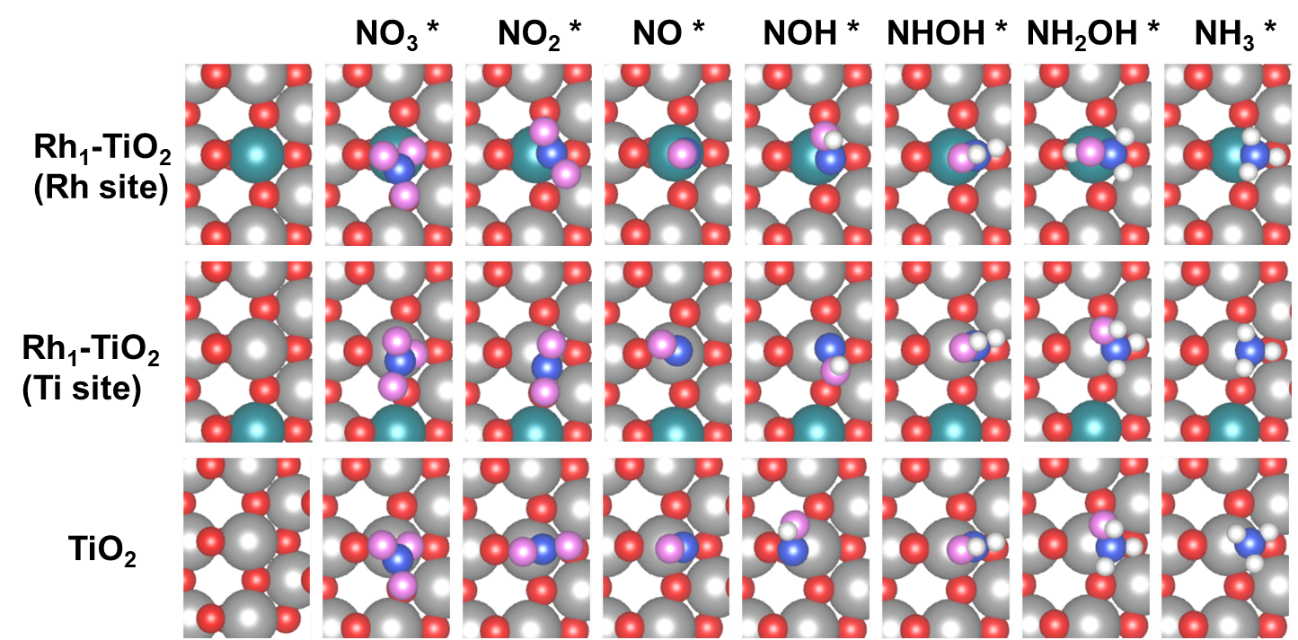


**Figure S21**. Atomic configuration of N-containing intermediates adsorbed on TiO_2_ and Rh and Ti site of Rh_1_-TiO_2_. Cyan, silver, white, and blue balls represent Rh, Ti, H and N atoms, red and purple balls represent O in TiO_2_ and N-containing intermediates, respectively.

**Table S1**. Elemental compositions obtained by ICP-OES of the as-prepared catalysts.

| Sample | Normalized atomic percentage (%) | | | | |
| --- | --- | --- | --- | --- | --- |
|  | Ti | Ru | Rh | Pd | Ir |
| Rh_1_-TiO_2_ | 98.7 | − | 1.08 | − | − |
| Rh_0.5_-TiO_2_ | 99.52 | − | 0.48 | − | − |
| Rh_2_-TiO_2_ | 98.04 | − | 1.96 | − | − |
| Ru_1_-TiO_2_ | 99.06 | 0.94 | − | − | − |
| Pd_1_-TiO_2_ | 98.88 | − | − | 1.12 | − |
| Ir_1_-TiO_2_ | 99.05 | − | − | − | 0.95 |

**Table S2**. Comparison of the surface parameters, mass activity and specific activity of electrocatalysts toward NO_3_RR.

| Samples | C_dl_  (mF cm^-2^) | R_f_^a^ | Surface area^b^  (m^2^ g_oxide_^-1^) | Mass activity^c^  (mA mg_Ru_ ) | Specific activity^c^ (mA cm_oxide_^-2^) |
| --- | --- | --- | --- | --- | --- |
| TiO_2_ | 1.59 | 26.48 | 33.1 | 405.3 | 1.23 |
| Rh_1_-TiO_2_ | 1.692 | 28.2 | 35.2 | 1573.1 | 4.47 |

*^a^*^:^ the R_f_ was calculated by dividing C_dl_ by the capacitance of ideal planar metal oxides with smooth surface, which was taken as 0.06 mF cm^-2^;

*^b^*^:^ the surface area was calculated by multiplying the electrode geometrical area by R_f_ and then normalized by taking into account the loading mass of electrocatalysts;

*^c^*^:^ the mass and specific activity were obtained from the current density values multiplying FE of NO_3_RR at -0.5 V vs RHE.

**Table S3**. Comparison of the recently reported Rh-based, TiO_2_-based and single atoms electrocatalysts for nitrate reduction.

| Catalyst | Electrolyte | NH_3_ yield rate | Faradaic efficiency | Ref. |
| --- | --- | --- | --- | --- |
| Rh_1_-TiO_2_ | 0.1 M KOH | 29.98 mg h^-1^ mg_cat_^-1^  at -0.5 V vs RHE | 94.7% | **This work** |
| Rh nanoflowers | 0.1 M Na_2_SO_4_+0.1 M KNO_3_ | 0.1 mg h^-1^ cm^-2^  at -0.1 V vs RHE | ~28% | ACS Catal. 2023, 13, 1513 |
|  |  | 0.2533 mg h^-1^ cm^-2^  at 0.2 V vs RHE | 95% |  |
| Rh nanoparticles | 0.1 M Na_2_SO_4_+0.1 M KNO_3_ | 0.026 mg h^-1^ cm^-2^  at -0.1 V vs RHE | ~22% |  |
| PA-RhCu | 0.1 M HClO_4_+0.05 M KNO_3_ | 2.40 mg h^-1^ mg_cat_^-1^  at 0.05 V vs RHE | 93.7% | Adv. Energy Mater. 2022, 12, 2103916. |
|  |  | 0.341mg h^-1^ mg_cat_^-1^  at -0.05 V vs RHE | 27.2% |  |
| RhNi bimetallenes | 0.1 M HClO_4_+0.05 M KNO_3_ | 13.4 mg h^-1^ mg_cat_^-1^  at 0.05 V vs RHE | 98.4 % | Adv. Mater. 2024, 2314351 |
|  |  | 3.9 mg h^-1^ mg_cat_^-1^  at 0.05 V vs RHE | 35.2% |  |
| Rh@Cu-0.6% | 0.1 M Na_2_SO_4_+ 0.1 M KNO_3_ | 21.61 mg h^-1^cm^-2^  at -0.4 V vs RHE | 93% | Angew. Chem. Int. Ed. 2022, e202202556 |
| TiO_2-x_ | 0.5 M Na_2_SO_4_/500 ppm NO_3_-N | 0.765 mg h^-1^ mg_cat_^-1^  at -1.6 V vs. SCE | 85% | ACS Catal. 2020, 10, 3533 |
| Pd/TiO_2_ | 0.25 M LiNO_3_+5 M LiCl | 0.112 mg h^-1^ mg_cat_^-1^  at -0.8 V vs. RHE | 92.1% | Energy Environ. Sci. 2021, 14, 3938 |
| Cu/Fe-TiO_2_ | 50 ppm NaNO_3_+0.5 M Na_2_SO_4_ | 8.5974 mg h^-1^ mg_cat_^-1^  at −1.4 V vs. SCE | 91.2% | Appl. Catal. B 2023, 325, 122360 |
| Ru1/TiOx | 1 M KOH+1 M NaNO_3_ | - | 87.3% | Angew. Chem.Int. Ed.2022, e2022082 |
| Co@TiO_2_ | 0.1 M PBS+0.1 M KNO_3_ | 6.3 mg h^-1^cm^-2^  at -0.7 V vs RHE | 96.7% | Small 2023, 19, 2208036 |
| Fe SACs | 0.1 M K_2_SO_4_+0.5 M KNO_3_ | 20 mg h^-1^ mg_cat_^-1^  at -0.85 V vs RHE | ~67% | Nat. Com. 2021, 12, 2870 |
| Fe-ppy SACs | 0.1 M KNO_3_+0.1 M HNO_3_ | 11.46 mg h^-1^ mg_cat_^-1^ at -0.7V vs RHE | Nearly 100% | Energy Environ. Sci. 2021,14, 3522 |

**Table S4**. Comparison of the recently reported electrocatalysts in alkaline electrolytes for nitrate reduction.

| Catalyst | Electrolyte | NH_3_ yield rate | Faradaic efficiency | Ref. |
| --- | --- | --- | --- | --- |
| Rh_1_-TiO_2_ | 0.1 M KOH | 29.98 mg h^-1^ mg_cat_^-1^  at -0.5 V vs RHE | 94.7% | **This work** |
| Ru/β-Co(OH)_2_ | 1 M KOH+1 M KNO_3_ | 24.12 mg h^-1^ mg_cat_^-1^  at  0.01 V vs. RHE | 98.78% | Energy Environ. Sci. 2023,16, 2483. |
| FePc/TiO_2_ | 0.1 M KOH+0.4 M KNO_3_ | 17.4 mg h^–1^cm^–2^  At –0.65 V vs RHE | 85% | Nat. Com. 2023, 14, 8036 |
| Fe-ppy SACs | 0.1 M KOH+0.1 M KNO_3_ | 11.46 mg h^-1^ mg_cat_^-1^ at -0.7V vs RHE | Nearly 100% | Energy Environ. Sci. 2021,14, 3522 |
| Co3O4/TM | 0.1 M KOH+0.1 M KNO_3_ | 12.67 mg h^–1^cm^–2^  At –0.6 V vs RHE | 95% | Small 2023, 19, 2303424 |
| Pd-Co3O4/TM | 0.1 M KOH+0.1 M KNO_3_ | 8.98 mg h^–1^cm^–2^  At –0.6 V vs RHE | 87% |  |
| RuCo@TDC | 0.1 M KOH+0.1 M KNO_3_ | 25.5 mg h^–1^cm^–2^  At –0.5 V vs RHE | ~93% | Journal of Colloid And Interface Science 668 (2024) 264–271 |
| Co@TDC | 0.1 M KOH+0.1 M KNO_3_ | 19.7 mg h^–1^cm^–2^  At –0.5 V vs RHE | ~90% |  |
| Fe-Ni2P | 1 M KOH+0.1 M KNO_3_ | 62 mg h^–1^cm^–2^  At –0.5 V vs RHE | 88% | Angew. Chem. Int. Ed. 2024, e202415300 |
| Ni2P | 1 M KOH+0.1 M KNO_3_ | 10 mg h^–1^cm^–2^  At –0.5 V vs RHE | 55% |  |
| Ru SASs/Co HNSs | 1 M NaOH+1 M NaNO_3_ | 10.2 mg h^–1^cm^–2^  At –0.5 V vs RHE | ~92% | Chemical Engineering Journal 490 (2024) 151883 |
| Co CHNWs@Co HNSs | 1 M NaOH+1 M NaNO_3_ | 9.3 mg h^–1^cm^–2^  At –0.5 V vs RHE | 90% |  |
| Cu-N4B2 | 1 M KOH+0.1 M KNO_3_ | 49.3 mg h^–1^cm^–2^  At –0.5 V vs RHE | 90% | **Energy Environ. Sci.**, 2024，10.1039/D4EE02746A |
| Cu-N4 | 1 M KOH+0.1 M KNO_3_ | 28.9 mg h^–1^cm^–2^  At –0.5 V vs RHE | 69% |  |

**Table S5**. Energy required for the elementary reactions involved in the nitrate reduction reaction on TiO_2_ and Rh and Ti site of Rh_1_-TiO_2_. The energy barrier of the rate-determining step has been highlighted in bold.

|  | elementary reaction | G_0_ (eV) | | |
| --- | --- | --- | --- | --- |
|  |  | TiO_2_ | Rh_1_- TiO_2_  (Rh site) | Rh_1_- TiO_2_  (Ti site) |
| 1 | *+NO_3_→NO_3_* | 1.19 | 0.11 | 0.85 |
| 2 | NO_3_*+H_2_O(g) →NO_2_*+2OH^−^ | -2.06 | -1.96 | -1.57 |
| 3 | NO_2_*+H_2_O(g) →NO*+2OH^−^ | -1.43 | -2.00 | -1.42 |
| 4 | NO*+H_2_O(g) →NOH*+OH^−^ | 0.80 | **0.74** | **0.97** |
| 5 | NOH*+H_2_O(g) →NHOH*+OH^−^ | -1.30 | -0.72 | -1.64 |
| 6 | NHOH*+H_2_O(g) →NH_2_OH*+OH^−^ | -1.45 | -1.77 | -1.50 |
| 7 | NH2OH*+H_2_O(g) →NH_3_*+2OH^−^ | -4.45 | -2.06 | -2.51 |
| 8 | NH_3_* →NH_3_+* | **1.23** | 0.21 | -0.63 |

**Supplementary References**

1. Mandlmeier, B.; Szeifert, J. M.; Fattakhova-Rohlfing, D.; Amenitsch, H.; Bein, T., Formation of interpenetrating hierarchical titania structures by confined synthesis in inverse opal. *Journal of the American Chemical Society* **2011,** *133* (43), 17274-82.

2. Kresse, G.; Furthmüller, J., Efficiency of ab-initio total energy calculations for metals and semiconductors using a plane-wave basis set. *Computational materials science* **1996,** *6* (1), 15-50.

3. Kresse, G.; Furthmüller, J., Efficient iterative schemes for ab initio total-energy calculations using a plane-wave basis set. *Physical review B* **1996,** *54* (16), 11169.

4. Perdew, J. P.; Burke, K.; Ernzerhof, M., Generalized gradient approximation made simple. *Physical review letters* **1996,** *77* (18), 3865.

5. Kresse, G.; Joubert, D., From ultrasoft pseudopotentials to the projector augmented-wave method. *Physical review b* **1999,** *59* (3), 1758.

6. Blöchl, P. E., Projector augmented-wave method. *Physical review B* **1994,** *50* (24), 17953.

7. Grimme, S.; Antony, J.; Ehrlich, S.; Krieg, H., A consistent and accurate ab initio parametrization of density functional dispersion correction (DFT-D) for the 94 elements H-Pu. *The Journal of chemical physics* **2010,** *132* (15).
